# Combination of Extrusion and Fermentation with *Lactobacillus plantarum* and *L. uvarum* Strains for Improving the Safety Characteristics of Wheat Bran

**DOI:** 10.3390/toxins13020163

**Published:** 2021-02-19

**Authors:** Elena Bartkiene, Egle Zokaityte, Vita Lele, Vytaute Starkute, Paulina Zavistanaviciute, Dovile Klupsaite, Darius Cernauskas, Modestas Ruzauskas, Vadims Bartkevics, Iveta Pugajeva, Zane Bērziņa, Romas Gruzauskas, Sonata Sidlauskiene, Antonello Santini, Grazina Juodeikiene

**Affiliations:** 1 Institute of Animal Rearing Technologies, Lithuanian University of Health Sciences, Tilzes g. 18, LT-47181 Kaunas, Lithuania; egle.zokaityte@lsmuni.lt (E.Z.); vita.lele@lsmuni.lt (V.L.); vytaute.starkute@lsmuni.lt (V.S.); paulina.zavistanaviciute@lsmuni.lt (P.Z.); dovile.klupsaite@lsmuni.lt (D.K.); darius.cernauskas@lsmuni.lt (D.C.); modestas.ruzauskas@lsmuni.lt (M.R.); sonata.sidlauskiene@lsmuni.lt (S.S.); 2 Department of Food Safety and Quality, Lithuanian University of Health Sciences, Tilzes g. 18, LT-47181 Kaunas, Lithuania; 3 Department of Anatomy and Physiology, Lithuanian University of Health Sciences, Tilzes g. 18, LT-47181 Kaunas, Lithuania; 4 Microbiology and Virology Institute, Lithuanian University of Health Sciences, Tilzes g. 18, LT-47181 Kaunas, Lithuania; 5 Institute of Food Safety, Animal Health and Environment BIOR, Lejupes iela 3, LV-1076 Riga, Latvia; vadims.bartkevics@bior.lv (V.B.); iveta.pugajeva@bior.lv (I.P.); zane.berzina@bior.lv (Z.B.); 6 Department of Food Science and Technology, Kaunas University of Technology, Radvilenu Rd. 19, LT-50254 Kaunas, Lithuania; romas.gruzauskas@ktu.lt (R.G.); grazina.juodeikiene@ktu.lt (G.J.); 7 Department of Pharmacy, University of Napoli Federico II, 80131 Napoli, Italy

**Keywords:** wheat bran, by-products, biosafety, extrusion, fermentation, chemical safety, mycotoxins

## Abstract

Processed wheat bran (W) is of great importance for food and feed. Consequently, the biosafety of W should be evaluated and improved with valorisation strategies. This study tested a design combining extrusion (at temperature of 115 and 130 °C; screw speeds of 16, 20, and 25 rpm) and fermentation with *Lactobacillus plantarum* and *L. uvarum* strains for the valorisation of W to provide safer food and feed stock. The influence of different treatments on biogenic amine formation, mycotoxin content, and free amino acids, as well as acidity, microbiological parameters, and sugar concentration, were analysed. This research showed that a combination of extrusion and fermentation with selected strains can change several aspects of W characteristics. There was a significant effect of applied treatments on acidity and the microbiological parameters of W, as well as biogenic amines content. The lowest total mycotoxin concentration (29.8 µg/kg) was found in extruded (130 °C; 25 rpm) and fermented with *L. uvarum* sample. Finally, the combination of the abovementioned treatments can be confirmed as a prospective innovative pre-treatment for W, capable of potentially enhancing their safety characteristics and composition.

## 1. Introduction

Globally, wheat production is more than 700 Mt/year—Million tons per year (FAOSTAT, 2014 and 2016, www.faostat.org), and of all wheat production, approximately one fifth is bran stock (90–150 Mt/year) [1], the main usage of which is as a feed supplement [2]. For the European Union (EU) economy, wheat and wheat by-products are of great importance for food and feed, as well as for biorefinery development [3]. For this reason, new strategies for the valorisation of wheat bran through its transformation into added-value stock are sought [4]. Wheat bran has a multi-layer structure [1]; however, at an industrial scale, different milling fractions are usually collected together [3]. Consumption of the wheat outer layer is associated with health benefits [5], and the modification of the wheat bran composition can lead to better technological properties as well as a higher functional value [6]. Reducing wheat bran particle size can lead to better accessibility of nutrients [7] and phenolic compounds possessing desirable antioxidant properties, inactivate several undesirable enzymes, and reduce biochemical reactivity [8]. Additionally, extrusion increases the solubility of wheat bran fibres depending on the extrusion parameters [9]. The most popular approach, wheat bran fermentation, has a great influence on the structure, biological activity, and bioavailability of wheat bran [10,11]. Additionally, fermentation with lactic acid bacteria (LAB) is considered an effective process to improve the sensory and nutritional quality of wheat bran [12]. However, current studies are mainly focused on the effects of LAB fermentation on sensory properties, technological characteristics, and antioxidant capacity; to date, changes in the broad spectrum of mycotoxins in wheat bran have not been analysed. However, this is very important because, despite the benefits associated with wheat bran consumption, safety concerns have been raised. Cereal grains, as well as wheat bran, can be frequently contaminated with *Fusarium* mycotoxins, *Alternaria* mycotoxins, and the ergot alkaloid groups [13]. Contaminations by aflatoxins (48%) and ochratoxin A (OTA) (14%), by *Aspergillus* species, and deoxynivalenol (DON) (21%) and fumonisins (13%), by *Fusarium* species, were reported in cereals [13]. Depending on their type and concentration, mycotoxins can cause adverse health effects in humans and animals [14,15]. *Alternaria* mycotoxins *(Alternaria alternata lycopersici*) has carcinogenic effect; aflatoxins by *Aspergillus* species are potential carcinogens, neurotoxins and immunosuppressants; DON and derivatives by *Fusarium* species can cause nausea, vomiting and stomach pains; chronic and fatal toxic effects; Enniatins by *Fusarium* species have antibiotic and ionophoric activity; Fumonisins by *Fusarium* species interfere with some steps that contribute to cell growth; OTA by *Aspergillus* species is a probable human carcinogen, neurotoxic and immunosuppressant; T-2 toxin and HT-2 toxin are the most toxic of the *Fusarium* trichothecenes and interferes with protein synthesis and DNA/RNA synthesis [13]. Meanwhile, mycotoxins can have cumulative effects at lower doses, resulting in chronic health effects [16,17]. Therefore, bio- and chemical safety is one of the biggest safety issues of cereal-derived products, which urgently needs attention, especially when the outer layer of cereals is used [18].

For these reasons, our hypothesis is that a combination of extrusion and fermentation with *Lactobacillus plantarum* and *L. uvarum* can lead to the improvement of the functional and safety characteristics of processed wheat bran (W) [19]. However, by using LAB fermentation, the formation of biogenic amines (BA) should be evaluated, because these low molecular weight organic bases can be formed as a result of normal LAB metabolic activity by the decarboxylation of wheat bran substrate amino acids [20]. LAB mainly produces histamine, tyramine and putrescine via decarboxylase or deiminase activities [21]. It was reported that putrescine and cadaverine could be formed by *Lactobacillus buchneri* strains, tyramine by several *Leuconostoc* strains, *Lactobacillus* and *Enterococci*, and histamine by *Streptococcus thermophilus* [21,22]. BA are important compounds in maintaining cells, as well as the proper viability of the body’s metabolic processes, such as protein synthesis, hormone synthesis, and DNA replication [23]. However, despite the positive effects on the body, physiological and toxicological effects (diarrhoea, food poisoning, vomiting, sweating or tachycardia) of BA, especially in high concentrations, make them undesirable compounds in food or feed [20,23]. In this study, to select the most appropriate technology, various parameters of processed and untreated wheat bran were analysed: acidity and microbiological parameters, concentration of sugars (fructose, glucose, sucrose, and maltose), free amino acid (FAA) content, and BA formation; the influence of the different treatments on the concentrations of 20 mycotoxins (alternariol; alternariol monomethyl ether; 17-dimethylaminoethylamino-17-demethoxygeldanamycin; 15-acetyldeoxynivalenol; deoxynivalenol; deoxynivalenol-3-glucoside; 15-acetoxyscirpenol; enniatin A and A1; fumonisin B1 and B2; meleagrin; sterigmatocystin; ochratoxin A and B; T-2 toxin; HT-2 toxin; fusarenone; neosolaniol; aflatoxin B1) were also evaluated.

## 2. Results and Discussion

### 2.1. Acidity and Microbiological Parameters of Wheat Bran

The acidity and microbiological parameters of processed wheat bran are shown in Table 1. Comparing the pH values of non-fermented and fermented samples, the greatest reduction in pH was found after 24 h of fermentation in non-extruded samples (W_ConLpl_ and W_ConLu_). Comparing extruded samples fermented with *L. plantarum* (24 h) with W_ConLpl_ (24 h), the pH of extruded fermented samples (W_ex115/Lpl_, W_ex130/16/Lpl_, W_ex130/20/Lpl_, W_ex130/25/Lpl_) was 9.2%, 15.2%, 11.7%, and 20.9% higher, respectively. Comparing extruded samples fermented with *L. uvarum* (24 h) with W_ConLu_ (24 h), the pH of extruded fermented samples (W_ex115/Lu_, W_ex130/16/Lu_, W_ex130/20/Lu_, W_ex130/25/Lu_) was 13%, 23.7%, 16.2%, and 21.4% higher, respectively. A very strong negative correlation was found between the samples’ pH and TTA (total titratable acidity (*r* = −0.957)) (Supplementary Material S1. Correlation coefficients). Comparing the concentration of L(+) and D(−) lactic acid isomers in fermented samples, the highest D(−)/L(+) ratio was found in W_ConLpl_ and W_ConLu_ samples (30.6 and 26.7, respectively). However, in extruded and fermented samples, the L(+) isomer concentration was increased, and in W_ex115/Lpl_, W_ex115/Lu_, and W_ex130/16/Lpl_ samples the D(−)/L(+) ratio was 1.4, 3.3, and 1.3, respectively. The predominant lactic acid isomer was L(+) in samples W_ex130/16/Lu_, W_ex130/20/Lpl_, W_ex130/20/Lu_, W_ex130/25/Lpl_, and W_ex130/25/Lu_. A moderate positive correlation was found between pH and L(+) isomer (*r* = 0.556); however, a very strong negative correlation was established between pH and D(−) isomer (*r* = −0.932) (Supplementary Material S1. Correlation coefficients). Very weak not significant positive correlations were found between TTA and L(+) and D(−) (*r* = 0.273 and *r* = 0.100, respectively) (Supplementary Material S1. Correlation coefficients). There was a significant effect of the type of LAB applied for fermentation and of extrusion (*p* ≤ 0.0001), as well as interaction of these factors (*p* ≤ 0.001), on pH, TTA, and L(+) and D(−) lactic isomer concentration in wheat bran samples (Supplementary Material Table S1).

During fermentation, monosaccharides are firstly fermented by LAB, generating organic acids which reduce the pH of the substrate [24]. This could explain why lower pH values were found in fermented non-extruded W. The changes in the ratio of lactic acid isomers could be related to the stereospecificity of lactate dehydrogenase enzymes and production conditions [25]. The L(+) isomer occurs naturally in the human body and is metabolized faster than the D(−) isomer. The latter is harmful because a higher amount in the body can lead to acidosis [26]. *L. plantarum* produces a racemic mixture of L(+) and D(−) isomers [27].

The lactic acid bacteria (LAB) count in fermented samples was, on average, 8.3 log_10_ CFU/g, and very strong negative and positive correlations were found between LAB count and pH, and between LAB count and TTA, respectively (*r* = −0.958 and *r* = 0.935, respectively) (Supplementary Material S1. Correlation coefficients). There was a significant effect of the type of LAB applied for fermentation and of extrusion, as well as interaction of these factors (*p* ≤ 0.0001), on the LAB count in wheat bran samples (Supplementary Material Table S1).

The mould/yeast (M/Y) count was similar in all extruded samples and did mostly not differ from the unprocessed wheat bran. However, in W_ConLpl_ samples, it was reduced by 14.6% and, in contrast, in W_ConLu_ samples it was increased by 12.2%, compared with non-fermented ones.

The lowest total bacteria count (TBC) was found in W_ex130/25_ samples (8.46 log_10_ CFU/g, respectively), and there was a significant effect of the type of LAB applied for fermentation (*p* = 0.0001) and of extrusion (*p* ≤ 0.0001), as well as the interaction of these factors (*p* = 0.003), on the TBC in wheat bran samples (Supplementary Material Table S1).

No culturable enterobacteria remained in fermented samples, and extrusion decreased the total enterobacteria count (TEC) in wheat bran samples; compared with non-extruded samples, the TEC was found, on average, to be 13.5%, 17.9%, 15.8%, and 24.1% lower in W_ex115_, W_ex130/16_, W_ex130/20_, and W_ex130/25_, respectively. There was a significant effect on the type of LAB applied for the fermentation and of extrusion, as well as interaction of these factors (*p* ≤ 0.0001), on the TEC in wheat bran samples (Supplementary Material Table S1).

The growth of LAB during W fermentation was influenced by nutrient availability and biophysical environmental factors (pH, water activity, and temperature). Carbon and energy are mainly obtained from carbohydrates and there are differences in sugar utilization between LAB strains [28]. However, other compounds, such as peptides, free amino acids, minerals, and free fatty acids, are also required for the optimal metabolism of LAB.

A high temperature during the extrusion process has a negative impact on microorganisms; that is why lower TEC was found in extruded samples. Moreover, particular LAB strains produce different inhibitory compounds (organic acids, hydrogen peroxide, free fatty acids, ethanol, etc.) to prevent contamination of fermented products with pathogenic bacteria, mould, or even yeast [29]. Our previous studies showed that *L. plantarum* and *L. uvarum* display antifungal and antimicrobial activity against the tested pathogenic bacteria and mould strains [19]. In the study of Arte et al. [30], no *Enterobacteriaceae* were found in wheat bran fermented with *L. brevis* E-95612 and *C. humilis* E-96250. Prücker et al. [31] reported that the growth of *Enterobacteriaceae* and yeasts/moulds is completely inhibited in wheat bran fermented with *L. plantarum*. However, it is also known that LAB and yeast can stimulate each other’s growth and cooperate in fermented foods. In sourdough, sucrose hydrolysis by *S. cerevisiae* stimulates *L. plantarum* growth, while *S. cerevisiae* may benefit from maltose hydrolysis by the mentioned LAB strain [32].

### 2.2. Influence of the Different Treatments on Fructose, Glucose, Sucrose, and Maltose Concentration in Processed Wheat Bran

The fructose, glucose, sucrose, and maltose concentrations in processed wheat bran are given in Table 1. As can be seen from the sugar analysis of W, W_Con_ contained monosaccharides, including glucose and fructose, while these monosaccharides were not found in almost all extruded non-fermented samples. Comparing non-fermented samples, fructose was found in W_Con_ and W_ex130/25_ samples (0.16 and 0.11 g/100 g, respectively). However, after fermentation, fructose was found in W_ex115/Lpl_, W_ex115/Lu_, W_ex130/16/Lpl_, W_ex130/20/Lpl_, and W_ex130/20/Lu_ samples (concentration, on average, 0.13 g/100 g). Glucose was found only in the group of non-extruded samples (concentration, on average, 0.66 g/100 g). Sucrose, in contrast, was found in all non-fermented extruded samples, its concentration ranging from 0.17 g/100 g (in W_ex115_) to 0.81 g/100 g (in W_ex130/25_). Sucrose was also found in one extruded fermented sample (W_ex130/16/Lu_). Comparing the maltose concentration in non-fermented samples, the highest concentration was found in W_ex115_ (0.48 g/100 g); the maltose concentration was, on average, 35.4%, 66.7%, 79.2%, and 77.1% lower in W_Con_, W_ex130/16_, W_ex130/20_, and W_ex130/25_ samples, respectively. Comparing the maltose content in non-fermented and fermented samples, in the non-extruded sample group the maltose concentration remained similar to that before fermentation; in the W_ex115_ sample group the maltose concentration was reduced, on average by 68.8% and 18.8% in samples W_ex115/Lpl_ and W_ex115/Lu_, respectively.

There was a significant effect of the type of LAB applied for the fermentation and of extrusion, as well as interaction of these factors (*p* ≤ 0.0001), on the fructose, glucose, sucrose, and maltose concentration in wheat bran samples (Supplementary Material Table S1). Additionally, we established a strong positive correlation between samples’ pH and sucrose concentrations (*r* = 0.653), strong negative correlations between TTA and sucrose concentration and between LAB count and sucrose concentration (*r* = −0.661 and *r* = −0.655, respectively), a moderate negative correlation between pH and fructose concentration (*r* = −0.411), a moderate positive correlation between TTA and fructose concentration (*r* = 0.413), and a weak positive correlation between LAB count and fructose concentration and weak positive non-significant correlation between pH and maltose concentration (*r* = 0.358 and *r* = 0.201, respectively), as well as weak negative non-significant correlations between pH and glucose concentration, TTA and maltose concentration, and LAB count and maltose concentration (*r* = −0.218, *r* = −0.258, and *r* = −0.257, respectively) (Supplementary Material S1. Correlation coefficients).

Carbohydrates in wheat bran consist of approximately 40% dietary fibre and 10% starch [3]. The changes in the content of the sugars analysed in treated W could be explained by the LAB activity and the effect of extrusion conditions (temperature, pressure, and mechanical shear). In fermented W, LAB consume simple sugars and can excrete endogenous enzymes for polysaccharide hydrolysis (sucrose) [10]. During the extrusion process, high temperature and shear may damage large molecules and improve the solubility of dietary fibre [9]. Due to this, higher amounts of lower molecular weight disaccharides and oligosaccharides can be formed and the magnitude of the effect may depend on screw speed and final temperature. Moreover, the decrease in the content of some reducing sugars (fructose, glucose, and maltose) in extruded samples could be an outcome of peculation due to the Maillard reaction [33].

### 2.3. Free Amino Acid and Biogenic Amine Content in Wheat Bran Samples

The FAA concentrations in cereal by-products are given in Table 2. Comparing the glutamine (Glu) concentration in non-fermented samples, extrusion at 130 °C and screw speeds of 20 and 25 rpm reduced the Glu concentration by 15.4%, and 18.3%, respectively, compared with non-extruded ones. Fermentation with *L. uvarum* increased the Glu concentration in W_ConLu_ and W_ex115/Lu_ samples, on average, by 14.6% and 9.9%, respectively. No asparagine (Asn) was found in wheat bran samples. Serine (Ser), histidine (His), and glycine (Gly) concentrations in non-fermented samples were, on average, 0.29, 0.12, and 0.27 g/100 g, respectively, and fermentation with both tested LAB strains had no influence on Ser, His, and Gly concentrations in samples. Similar tendencies were found for threonine (Thr) and arginine (Arg) as for the previously mentioned FAA: in non-fermented samples, their concentrations were, on average, 0.27 and 0.31 g/100 g, respectively, and fermentation was not a significant factor for the Thr and Arg concentration in samples. The alanine (Ala) concentration in non-fermented samples ranged from 0.21 to 0.31 g/100 g, in W_ex130/25_ and W_ex115_ samples, respectively. Comparing the Ala concentration in fermented samples, it was not changed in any extruded samples after fermentation, however, in W_ConLpl_ samples in which it increased (on average by 17.2%). The tyrosine (Tyr) concentration in non-fermented samples was, on average, 0.19 g/100 g, and fermentation was not a significant factor for Tyr concentration in samples fermented with both LAB strains. The cysteine (Cys) concentration in non-fermented samples ranged from 0.34 g/100 g (in W_Con_) to 0.40 g/100 g (in W_ex115_ and W_ex130/20_). Comparing fermented and non-fermented samples, 22.7% and 18.2% higher Cys concentrations were found in W_ConLu_ and W_ex130/16/Lu_, respectively, compared with non-fermented samples in the same group. Comparing the valine (Val) concentration in non-fermented samples, the highest Val content was found in W_ex115_ samples (0.34 g/100 g); however, after fermentation, the Val concentration increased in W_ConLpl_ and W_ConLu_ samples (on average 0.34 g/100 g), and in other fermented samples no changes in Val concentration were found. Concentrations of methionine (Met), tryptophan (Trp), phenylalanine (Phe), isoleucine (Ile), leucine (Leu), and lysine (Lys) in non-fermented samples were similar between the different groups; however, after fermentation, Trp and Phe concentrations were increased (in W_ex130/16/Lu_ by 23.8% and 31.3%, respectively). Comparing the proline (Pro) concentration in non-fermented samples, it was found that, by increasing extrusion temperature and screw speed, the Pro concentration in samples was reduced, the lowest Pro content being found in W_ex130/20_ and W_ex130/25_ samples (on average, 0.28 g/100 g). In W_Con_, W_ex115_, and W_ex130/16_ samples, the concentrations of Pro were 1.7-, 2.9-, and 3.8-times higher, respectively, than those of the W_ex130/20_ and W_ex130/25_ samples. Comparing non-fermented samples with fermented ones, an increase in Pro concentration was found after fermentation in non-extruded samples (increased 2.3 and 2.2 times in W_ConLpl_ and W_ConLu_ samples, respectively), as well as in W_ex115/Lu_ samples (increased 1.3 times). However, in other fermented samples, the Pro concentration was the same, or lower, than that in non-fermented ones.

Finally, the fermentation of non-extruded, as well as extruded, wheat bran led to increases in some FAA: in W_ConLpl_—Ala, Val, and Pro, in W_ConLu_—Glu, Cys, Val, and Pro, in W_ex115/Lu_—Glu, and Pro, in W_ex130/25/Lpl_—Lys, and in W_ex130/16/Lu_—Cys and Phe.

Protein content in wheat bran can reach approximately 18% [3]. The changes in the FAA content of fermented W could be related to the fact that LAB excrete endogenous proteases and promote protein hydrolysis in fermented substrate [34]. Besides that, particular amino acids and peptides are required for LAB metabolism. The results obtained for FAA are in agreement with the study of Zhao, Guo, and Zhu [35], who also observed an increase in the total FAA content of wheat bran after fermentation with LAB. Furthermore, alterations in the samples’ FAA content might be also influenced by extrusion conditions, including temperature and moisture. Generally, extrusion induces chemical and physical changes in protein-rich materials [36]. Increased temperature during extrusion favours the Maillard reaction and can reduce the quality and quantity of most amino acids [33]. It has been reported that extrusion mostly affects Lys, Cys, and Arg [37].

The BA content in all wheat processing by-product samples is given in Table 3. No phenylethylamine (PHE), tyramine (TYR), or spermidine (SPRMD) were found in wheat bran samples; cadaverine (CAD) was found in three sample types (W_Con_ and W_ex130/25/Lu_: on average 33.8 mg/kg; W_ConLpl_: on average 48.9 mg/kg), and histamine (HIST) in two sample types (on average 9.2 mg/kg in W_Con_ and W_ex130/25/Lu_). Comparing the putrescine (PUT) concentration in non-fermented samples, it was, on average, 13.1%, 32.0%, 39.4%, and 14.7% higher in extruded samples than in non-extruded ones. Comparing the PUT concentration in fermented and non-fermented samples, it depended on extrusion conditions, as well as on the LAB strain used for fermentation. Increased PUT concentrations were found in W_ConLu_, W_ex115/Lpl_, W_ex115/Lu_, and W_ex130/25/Lpl_ samples (16.5%, 24.2%, 24.7%, and 33.2% higher, respectively, than non-fermented samples in the same group). Opposite effects were found for PUT concentration in W_ex130/16/Lpl_, W_ex130/16/Lu_, W_ex130/20/Lpl_, and W_ex130/25/Lu_ samples, in which the PUT concentration was reduced after fermentation, on average by 14.0%, 9.0%, 15.9%, and 14.7%, respectively, compared with non-fermented samples in the same group. Nonetheless, almost all processing procedures enhanced the PUT concentration (compared to W_Con_). Comparing the spermine (SPRM) concentration in non-fermented samples, some effects were found—by increasing the extrusion temperature and screw speed, the SPRM concentration in samples was reduced, the lowest being found in W_ex130/25_ samples (25.3 mg/kg). Comparing the SPRM concentration in non-fermented and fermented samples, no significant differences were found in most of the samples; however, 11.4% and 27.0% lower SPRM concentrations were found in W_ConLpl_ and W_ConLu_, respectively, compared with non-fermented samples. However, in W_ex115/Lpl_, W_ex130/25/Lpl_, and W_ex130/25/Lu_ samples, the SPRM concentration increased after fermentation, on average, by 20.4%, 24.7%, and 29.5%, respectively, compared with non-fermented samples. Finally, in most of the treated samples, compared with control samples, no CAD or HIST remained, the exceptions being W_ConLpl_ and W_ex130/25/Lu_. Additionally, the fermentation of wheat bran samples extruded at 130 °C with a screw speed of 16 rpm with *L. plantarum* and *L. uvarum* led to a reduction in PUT, without the other BA increasing. The same tendencies were also found for wheat bran samples extruded at 130 °C with a screw speed of 20 rpm fermented with *L. plantarum*.

The presence of BA in food is associated with natural formation as physiological compounds or synthesis by microorganisms through the decarboxylation of FAA [38]. The variety and concentration of BA depend on the materials’ chemical compositions, the decarboxylase activity of microorganisms, and processing conditions. According to the FDA and EFSA, the consumption of high levels of HIS and TYR can elicit food poisoning, while other BA usually cause food allergies [22]. Specific legislation only covers histamine in fishery products and no criteria have been established for other BAs or other food products, such as meat, dairy, or other products, despite the presence of important levels of BA in all types of food and the potential health risk in certain sectors of society where these products are consumed [38]. Particular LAB are able to produce BA from amino acids in fermented foods. In our research, HIST, TYR, SPRMD, PHE, and CAD were not found or found only in several samples in small amounts. It has been reported that the polyamine PUT, which was abundant in all tested W, can be produced from Arg or ornithine and further used for the formation of SPRM [39]. PUT is a common BA in food, but the data regarding its toxicity are scare. The research of del Rio [40] showed that PUT is cytotoxic at concentrations found in BA-rich foods and can cause cell necrosis but does not induce apoptosis. However, no human dose-response information is provided but negative impact on human health, such as cardiovascular diseases, is known. The toxicity of BA depends on synergistic effects, e.g., HIST toxicity is enhanced by the presence of CAD, PUT, and TYR [41].

### 2.4. Influence of the Different Treatments on Mycotoxin Concentration in Wheat Bran

The mycotoxin concentrations in processed wheat bran are shown in Table 4. Comparing the alternariol (AOH) concentration in non-fermented samples, the lowest was found in W_ex115_ (0.85 µg/kg). In the non-fermented samples, the AOH concentration was, on average, 2.1, 1.2, 2.1, and 3.9 times higher (in W_Con_, W_ex130/16_, W_ex130/20_, and W_ex130/25_, respectively), compared to W_ex115_. Comparing AOH in fermented and non-fermented samples, a lower or similar AOH concentration was found in most of the fermented samples compared with non-fermented samples, except for W_ex130/20/Lu_, in which the AOH concentration was 1.8 times higher than in non-fermented samples in the same group.

Comparing the alternariol monomethyl ether (AME) concentration in non-fermented samples, no AME was found in W_Con_, W_ConLpl_, W_ConLu_, W_ex115_, W_ex130/16/Lpl_, W_ex130/16/Lu_, or W_ex130/20/Lpl_; however, in samples extruded at the higher temperature with highest screw speed, its concentration was 3.45 µg/kg, and it decreased after fermentation with both LAB strains, on average, by 2.9 times.

17-dimethylaminoethylamino-17-demethoxygeldanamycin (17-DMAG) was found only in samples extruded at the higher temperature (130 °C), with higher levels in samples extruded at higher screw speed (20/25 rpm vs 16 rpm). Moreover, a higher concentration of 17-DMAG was found in W_ex130/20/Lu_ and W_ex130/25/Lpl_ samples (on average by 29.5% and 21.9%, respectively) compared with non-fermented samples in the same group.

Comparing non-fermented samples, the highest concentration of deoxynivalenol (DON) was found in non-extruded samples (58.8 µg/kg); in extruded samples (W_ex115_, W_ex130/16_, W_ex130/20_, and W_ex130/25_) the DON concentration was, on average, 23.3%, 44.0%, 58.5%, and 60.5% lower, respectively. In most cases, the DON concentration showed a tendency to reduce after fermentation with both LAB strains (except W_ex115/Lu_, in which it increased by 14.9%), and the greatest reduction was found in non-extruded fermented samples (reduced, on average, by 2.2 times).

Comparing the 15-acetyldeoxynivalenol (15-DON) concentration in non-fermented samples, the lowest 15-DON concentration was found in non-extruded samples (50.18 µg/kg); it was 33.2% higher in W_ex115_, 52.9% higher in W_ex130/16_, 41.4% higher in W_ex130/20_, and 39.9% higher in W_ex130/25_. Comparing the 15-DON concentration in non-fermented and fermented samples, fermentation reduced it in all cases, and no 15-DON was found in W_ex130/16/Lu_, W_ex130/20/Lu_, or W_ex130/25/Lu_ samples.

Comparing non-fermented samples, the highest concentration of deoxynivalenol-3-glucoside (D3G) was found in non-extruded samples (in W_Con_: 3.93 µg/kg); in extruded samples (W_ex115_, Wex_130/16_, W_ex130/20_, and W_ex130/25_), compared to W_Con_, the D3G content was, on average, 29.0%, 53.4%, 73.9%, and 71.8% lower, respectively. In most of the fermented samples, the D3G concentration was lower than in non-fermented samples in the same group; the exception was W_ex115/Lpl_, in which the D3G concentration was similar to that before fermentation.

Comparing the meleagrin (MEL) concentration in non-fermented samples, extrusion at 130 °C reduced it in all cases, and in W_ex130/16_, W_ex130/20_, and W_ex130/25_ samples, the MEL concentration was, on average, 36.7%, 46.7%, and 83.3% lower, respectively, compared with the control samples. However, in W_ex115_ samples, a higher concentration of MEL was found compared with non-extruded samples (30.2% higher). Comparing non-fermented and fermented samples, the MEL concentration was reduced in most of the fermented samples, and no MEL was found in W_ex130/20/Lu_ samples.

No neosolaniol (Neo) was found in non-extruded samples (non-fermented and fermented) or in W_ex130/16/Lpl_ samples. After fermentation, the Neo concentration showed a tendency to reduce, on average, two fold.

No 15-acetoxyscirpenol (15ACS) was found in the non-fermented samples, W_ex130/16_ and W_ex130/25_; however, after fermentation with *L. plantarum* and *L. uvarum,* the 15ACS concentration was increased in this group of samples, on average, to 1.66 µg/kg, respectively. In all cases, the 15ACS concentration in samples increased after fermentation, and the highest was found in non-extruded fermented samples (on average, 20.6 µg/kg).

Comparing the enniatin A (ENN A) concentration in non-fermented samples, the highest concentration of ENN A was found in W_Con_ samples (5.31 µg/kg); in extruded non-fermented samples, the ENN A content was, on average, 1.38 µg/kg. However, after fermentation, the ENN A concentration increased in all extruded samples (on average by 24.5, 15.9, 8.6, 7.6, 1.7, 1.7, and 1.2 times in W_ex115/Lpl_, W_ex115/Lu_, W_ex130/16/Lpl_, W_ex130/16/Lu_, W_ex130/20/Lpl_, W_ex130/20/Lu_, W_ex130/25/Lpl_, and W_ex130/25/Lu_; the exception was W_ex130/25/Lu_ in which the ENN A concentration remained similar).

Comparing the enniatin A1 (ENN A1) concentration in non-fermented samples, a lower ENN A1 concentration was found in all extruded samples, on average by 4.4 times. Opposite tendencies were found in non-extruded and extruded samples after fermentation, the ENN A1 content being reduced in non-extruded fermented samples; however, in extruded fermented samples, the ENN A1 concentration was increased in all cases, the highest being found in W_ex115/Lpl_ and W_ex115/Lu_ samples (9.65 and 6.22 µg/kg, respectively).

Fumonisin B1 (FB1) was found in two groups of samples: non-fermented and fermented W_ex130/20_ and W_ex130/25_, and the FB1 concentration was reduced in both groups of samples (except W_ex130/25/Lu_) after fermentation (on average, by 2 times). Fumonisin B2 (FB2) was found just in one sample (W_ex130/20_: 0.07 µg/kg).

No sterigmatocystin (STC) was found in W_Con_ and W_ConLpl_ samples; however, in other samples, the STC concentration ranged from 0.09 to 2.11 µg/kg (in W_ConLu_ and W_ex130/20/Lpl_, respectively).

Fusarenon X (FUSX) was found in all non-fermented samples and in just one fermented sample (We_x130/20/Lu_). Additionally, comparing the FUSX concentration in non-extruded and extruded samples, it was, on average, 1.7 times lower in extruded samples.

Comparing non-fermented samples, the lowest T-2 toxin (T-2) concentration was found in non-extruded samples (0.18 µg/kg); in extruded samples the T-2 concentration ranged from 0.85 to 1.48 µg/kg (in W_ex130/20_ and W_ex130/25_, respectively). Comparing non-fermented and fermented samples, fermentation with *L. uvarum* eliminated T-2 from the samples in all cases; however, in W_ConLpl_, W_ex115/Lpl_, and W_ex130/16/Lpl_ samples, the T-2 concentration was increased (on average, by 9.4, 2.9, and 2.5 times).

Comparing non-fermented samples, the HT-2 toxin (HT-2) was found only in W_Con_ and W_ex115_ samples (3.76 and 2.85 µg/kg, respectively), and after fermentation its content was reduced. However, after fermentation, HT-2 was found in W_ex130/16/Lpl_, W_ex130/16/Lu_, W_ex130/20/Lpl_, W_ex130/20/Lu_, W_ex130/25/Lpl_, and W_ex130/25/Lu_ samples (on average, 0.93 µg/kg).

Comparing non-fermented samples, a lower ochratoxin A (OTA) concentration was found in extruded samples in all cases, and after fermentation, no OTA was found in most of the samples (the exceptions being W_ex115/Lpl_ and W_ex130/16/Lu_). Ochratoxin B (OTB) was found in just two fermented samples (W_ex115/Lpl_ and W_ex115/Lu_, on average 0.08 µg/kg).

Comparing non-fermented samples, the highest concentration of aflatoxin B1 (AFB1) was found in non-extruded samples (in W_Con_: 3.20 µg/kg); in _Wex115_, W_ex130/16_, W_ex130/20_, and W_ex130/25_ samples the concentration was, on average, 20.3%, 46.9%, 71.6%, and 71.3% lower, respectively. No AFB1 was found in fermented samples.

ANOVA indicated that there was a significant effect of the type of LAB applied for the fermentation and of extrusion, as well as interaction of these factors, on all the analysed mycotoxin concentrations in wheat processing by-product samples (*p* ≤ 0.001) (Supplementary Material Table S1). Comparing the total identified mycotoxin concentration in samples, a lower mycotoxin content was found in most of the extruded and extruded/fermented samples (except in W_ex130/16_ and W_ex115/Lpl_): in W_ConLpl_ by 40.2%, W_ConLu_ by 40.0%, W_ex115_ by 8.9%, W_ex115/Lu_ by 6.9%, W_ex130/16/Lpl_ by 49.7%, W_ex130/16/Lu_ by 64.6%, W_ex130/20_ by 17.2%, W_ex130/20/Lpl_ by 65.8%, W_ex130/20/Lu_ by 73.8%, W_ex130/25_ by 17.7%, W_ex130/25/Lpl_ by 68.6%, and in W_ex130/25/Lu_ by 80.6%. Finally, the most effective treatments for reducing the mycotoxin content in wheat bran were W_ex130/25/Lu_ (total mycotoxin content 29.8 µg/kg), W_ex130/20/Lu_ (40.1 µg/kg), W_ex130/25/Lpl_ (48.2 µg/kg), W_ex130/20/Lpl_ (52.5 µg/kg), and W_ex130/16/Lu_ (54.4 µg/kg).

Multiple mycotoxins in feed and food have been recognized by European regulatory bodies as emerging risks in food safety and security with regards to animal and human health [42,43]. European Commission (EC) maximum permitted levels for aflatoxin B1 are 2 and 0.1 µg/kg (for direct human consumption and children/infants, respectively) in cereals and cereal based products; for DON—500 and 200 µg/kg; for enniatins—800 µg/kg in maize-based breakfast cereals/snacks; for fumonisins—800 and 200 µg/kg in maize-based breakfast cereals/snacks; for OTA—3 and 0.5 µg/kg; T-2 and HT-2—100–200 µg/kg and 50 µg/kg) [13]. The application of extrusion or extrusion/fermentation to W resulted in a lower mycotoxin content in most of the samples and these levels were lower than permitted by EC for human consumption (except for AFB1 in W_ex115_ and for OTA in W_ex130/16/Lu_). In accordance with our results, other researchers have observed similar mycotoxin detoxification results. Approximately 48% of zearalenone was degraded by *L. plantarum* in 48 h [44]. The study of Kaushik [45] revealed that very high roasting and extrusion temperatures are needed to bring a large reduction in mycotoxin concentrations, approaching acceptable background levels. Chlebicz and Slizewska [46] studied the effect of probiotic bacteria from the genus *Lactobacillus* on mycotoxin detoxification and reported high detoxification rates for aflatoxin B1, T-2, and zearalenone, with the concentration reduced, on average, by 60%, 61%, and 57%, respectively. Reductions of 100%, 95% and 83% for fumonisins, aflatoxins and zearalenone, respectively, have been reported during the extrusion cooking of cereals, while lower reductions were observed for deoxynivalenol, ochratoxin A and moniliformin, where maximum reductions did not exceed 55%, 40% and 30%, respectively [47]. Samar et al. (2001) found that DON within the wheat bread-making process might reduce DON levels during the fermentation stages from 41% to 56% [48]. The higher temperature of the extrusion process has a greater capability to degrade mycotoxins like OTA. Scudamore et al. (2004) found that the level of OTA reduced till 40% upon the increase in temperature from 100 to 150 °C, however further increasing in temperature was not significant on the OTA content [49]. It was reported 33% decrease in the OTA concentration during the dough fermentation [50].

## 3. Conclusions

To our knowledge, this is the first study based on the use of a combination of extrusion and fermentation with *L. plantarum* and *L. uvarum* strains for wheat bran valorisation. It was indicated that appropriate extrusion parameters and LAB strain selection led to a higher L(+) isomer formation and lower TEC in wheat bran. The strongest correlations were found between samples’ pH, TTA, and LAB count, respectively, and sucrose concentration. Fermentation increased Ala, Val, and Pro in W_ConLpl_, Glu, Cys, Val, and Pro in W_ConLu_, Glu, and Pro in W_ex115/Lu_, Cys, and Phe in W_ex130/16/Lu_, and Lys in W_ex130/25/Lpl_. A lower mycotoxin content was found in most of extruded and extruded/fermented W. The application of extrusion or extrusion/fermentation to W resulted in a lower mycotoxin content in most of the samples and these levels were lower than permitted by EC for human consumption (except for AFB1 in W_ex115_ and for OTA in W_ex130/16/Lu_). The type of LAB applied for fermentation and extrusion had a significant impact on the PUT, CAD, HIST and SPRM concentration in wheat bran samples. Finally, a combination of extrusion and fermentation with *L. plantarum* and *L. uvarum* strains can be confirmed as a prospective innovative pre-treatment for wheat bran, capable of potentially enhancing its composition and safety characteristics.

## 4. Materials and Methods

### 4.1. Processing of Wheat Bran

Wheat bran (nonprocessed and extruded) was obtained from the SME “Ustukiu malunas” (Pasvalys, Lithuania). Wheat bran was extruded at this enterprise at industrial scale at the different temperatures (Parallal Twin Screw Extruder DKM-EII75x28A, Dekuma, Dongguan, Guangdong, China; double-screw). Temperature in different extrusion zones was I-60–61 °C, II-70 °C and III-90 °C, moisture content 20%, feed rate F—8.2 kg/h, diameter of the nozzle—6 mm. Moisture of the final wheat bran samples (after extrusion) was 11%. Four different treated wheat bran sample groups were prepared (W_ex115_—extruded at 115 ° C, speed of the screw—16 rpm; W_ex130/16_—extruded at 130 °C, speed of the screw—16 rpm; W_ex130/20_—extruded at 130 °C, speed of the screw—20 rpm; W_ex130/25_—extruded at 130 °C, speed of the screw—25 rpm). Nonextruded wheat bran samples group was used as a control (W_Con_).

The lactic acid bacteria (LAB) strains *L. plantarum* LUH 122 and *L. uvarum* LUH 24 were used for extruded and nonextruded wheat bran fermentation. The LAB strains were obtained from the Department of food safety and quality at the Lithuanian University of Health Sciences (Kaunas, Lithuania). From the previous studies it was known that the abovementioned strains possess antimicrobial activities against various pathogenic and opportunistic strains, as well as antifungal activities [19,51]. The LAB strains, before an experiment, were stored at −80 °C (Microbank system, Pro-Lab Diagnostics, Birkenhead, UK) and multiplied in de MRS broth (Man-Rogosa-Sharpe, CM 0359, Oxoid Ltd., Hampshire, UK) at 30 ± 2 °C for 48 h, before use for the fermentation of wheat bran. The wheat by-products, water and a suspension of LAB strain (3% from dry matter of the wheat bran mass) containing 8.9 log_10_ CFU/mL, were fermented at 30 ± 2 °C for 24 h. For 100 g of wheat bran, 60 mL water was used.

**Figure 1:**
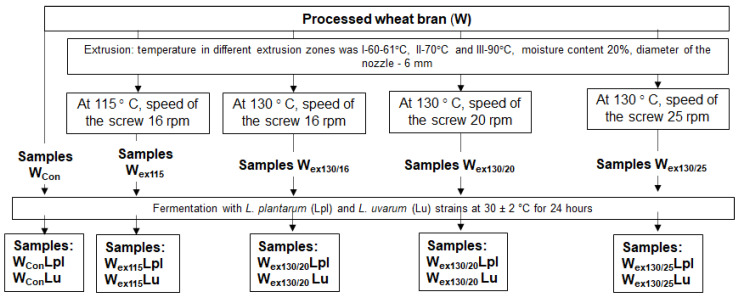
Principal scheme of samples preparation.

Finally, ten fermented wheat bran samples groups were prepared (from nonextruded wheat bran: W_Con_Lpl, W_Con_Lu; from extruded at 115 °C, speed of the screw 16 rpm fermented wheat bran samples: W_ex115/Lpl_, W_ex115/Lu_; extruded at 130 °C, speed of the screw 16 rpm fermented wheat bran samples: W_ex130/16/Lpl_, W_ex130/16/Lu;_ extruded at 130 °C, speed of the screw 20 rpm fermented wheat bran samples: W_ex130/20/ Lpl_, W_ex130/20/Lu;_ extruded at 130 °C, speed of the screw—25 rpm fermented wheat bran samples: W_ex130/25/Lpl_, W_ex130/25/Lu_). Principal scheme of samples preparation is shown in Figure 1. Each processing procedure was performed once, followed by analyses of 5 subsamples each.

### 4.2. Evaluation of Acidity and Microbiological Characteristics of the Wheat Bran Samples

The pH was measured using a pH electrode (PP-15; Sartorius, Goettingen, Germany). The total titratable acidity (TTA) was evaluated for a 10 g sample of sample mixed with 90 mL of water, and the results were expressed in mL of 0.1 mol/L NaOH solution required to achieve a pH value of 8.2. For L-(+) and D-(−) lactic acid isomers concentration evaluation, a specific Megazyme D-/L-Lactic Acid (D-/L-Lactate) (Rapid) Assay Kit (Megazyme Int., Vienna, Austria) was used. The determination of LAB, total bacteria (TBC), enterobacteria (TEC), and mould/yeast (M/Y) counts in samples was performed according to Bartkiene et al. [52]. The limit of detection (LOQ) for lactic acid content—0.02 g/100 g sample. The limit of detection (LOQ) for total enterobacteria count—1 CFU/g.

### 4.3. Analysis of the Sugars in Processed Wheat Bran

For determination of sugars concentration, 2–3 g of sample were diluted with ~70 mL of distilled/deionized water, heated to 60 °C in a water bath for 15 min, clarified with 2.5 mL Carrez I (85 mM K_4_[Fe(CN)_6_] × 3H_2_O) and 2.5 mL Carrez II (250 mM ZnSO_4_ × 7H_2_O) solutions, and made up to 100 mL with distilled/deionized water. After 15 min, the samples were filtered through a filter paper and a 0.22 μm nylon syringe filter before analysis. A 2 mg/mL standard solution of sugars mixture (Sigma Aldrich, Schnelldorf, Germany) was prepared following dilution with distilled/deionized water.

Sugars concentration analysis was performed by Ultra Performance Liquid Chromatography (UPLC). The UPLC apparatus was a Shimadzu LC-20AD (Shimadzu Corp., Kyoto, Japan) equipped with Evaporative Light Scattering Detector ELSDLTII (Shimadzu Corp., Kyoto, Japan) detector. Chromatographic conditions were as follows: the eluent was a mixture of 75 parts by volume of acetonitrile and 25 parts by volume water, flow rate was 1.2 mL/min, 20 μL was injected. The YMC-Pack Polyamine II 250 × 4.6 mm, 5 μm (YMC Co., Ltd., Kyoto 600-8106, Japan) column was used. Column temperature was set at 28 °C. The limit of detection (LOQ) for sugars—0.01 g/100 g sample.

### 4.4. Determination of Free Amino Acids and Biogenic Amines Content in Wheat Bran Samples

Free amino acids (FAA) were analysed by GC-FID instrument (Agilent 6890N, California, USA) with flame ionization detection after an ion-exchange solid phase extraction and chloroformate derivatization using EZ:faast technology (Phenomenex, Canada, USA). Standard solutions of the amino acids aspartic acid (Asp), alanine (Ala), glycine (Gly), valine (Val), leucine (Leu), isoleucine (Ile), threonine (Thr), serine (Ser), proline (Pro), asparagine (Asn), methionine (Met), glutamine (Glu), phenylalanine (Phe), lysine (Lys), histidine (His), arginine (Arg), tyrosine (Tyr), tryptophan (Trp), and cysteine (Cys) were used, in addition to the internal standard DL-Norvaline (NVAL). The analysis is detailed in [53].

The extraction and determination of biogenic amines (BA) in wheat samples followed the procedures developed by Ben-Gigirey et al. [54] with some modifications [55]. Perchloric acid (0.4 mol/L, 10 mL) containing a known amount of 1.7-diamino-heptane was used for BA extraction. A 5-(dimethylamino)naphtha-lene-1-sulphonyl chloride (dansyl chloride reagent) (10 mg/mL, 2 mL) was used for sample derivatization. The chromatographic analyses were carried out using a Varian ProStar HPLC system (Varian Corp., Canada, USA) with a ProStar 325 UV/VIS Detector, and Galaxy software (Agilent, California, USA) for data processing. A Discovery^®^ HS C18 column (150 × 4.6 mm, 5 µm; SupelcoTM Analytical, Bellefonte, PA, USA) was used. Ammonium acetate (0.1 mol/L) and acetonitrile were used as the mobile phases by a flow rate of 0.45 mL/min.

The limit of detection (LOQ) for biogenic amines concentration—0.1–0.6 mg/kg, for free amino acid—0.01–0.1 mg/100 g. Size of subsamples—5.

### 4.5. High-Performance Liquid Chromatography Coupled to Triple Quadrupole Mass Spectrometry (HPLC- MS/MS) for Mycotoxin Analysis

The following mycotoxins were analysed in samples: alternariol; alternariol monomethyl ether; 17-dimethylaminoethylamino-17-demethoxygeldanamycin; 15-acetyldeoxynivalenol; deoxynivalenol; deoxynivalenol-3-glucoside; 15-acetoxyscirpenol; enniatin A and A1; fumonisin B1 and B2; meleagrin; sterigmatocystin; ochratoxin A and B; T-2 toxin; HT-2 toxin; fusarenone; neosolaniol; aflatoxin B1.

Sample preparation. Samples (5.00 ± 0.01 g) were accurately weighed in 50 mL PP tubes. The quality control (blank) samples were supplemented with mycotoxin standard solutions at the appropriate spiking levels. Then water (10 mL), acetonitrile (10 mL), and formic acid (20 µL) were gradually added to the tubes and extraction was started by mixing for 10 min on mechanical shaker. One portion of the QuEChERS buffer salt kit was added to each of the tubes and the extraction was continued for additional 10 min. The obtained mixtures were centrifuged (1313× *g*, 5 min) and the supernatants were transferred to 15 mL centrifuge tubes and stored for 15 min at −80 °C in a Heto PowerDry^®^ freeze dryer (Thermo Fisher Scientific, Loughborough, UK). After removal, the extracts were immediately centrifuged (2626× *g*, 5 min) at 10 °C. Then, 2500 µL of extracts were transferred to 15 mL PP tubes and were evaporated to dryness at 50 °C under a gentle nitrogen stream. The dry residues were reconstructed in 100 µL of 50% acetonitrile in water with 0.1% formic acid and shaken 20 min. Then diluted by 400 µL water with 0.1% formic acid (total dilution factor = 5) and shaken for 10 min. Extracts were filtered through centrifuge filters (3900× *g*, 10 min) and transferred into the autosampler for analysis.

Chromatographic method. The analysis was performed on an UltiMate™ 3000 (Thermo Fisher Scientific, Loughborough, UK) HPLC coupled with a Thermo Scientific TSQ Quantiva MS/MS detector. The separation was performed on a Phenomenex Luna C18 reversed-phase analytical column (150 × 2.0 mm, 3 µm). The autosampler was maintained at 4 °C and the column temperature was 40 °C. The sample injection volume was 40 µL. Ion monitoring was conducted in both positive and negative ion modes and the mass analysis was performed in selected reaction monitoring (SRM) mode. The following instrumental settings were used: spray voltage 3.5 kV (positive ion mode), 2.5 kV (negative ion mode), vaporiser temperature 350 °C, ion transfer temperature 300 °C, sheath gas 55 arbitrary units (arb), auxiliary gas 25 arb, and sweep gas 5 arb. Data processing was performed with Xcalibur™ software (Thermo Fisher Scientific, Loughborough, UK). For more detailed information, see Additional Information in Supplementary Material.

### 4.6. Statistical Analysis

The results were expressed as the mean (*n* = 5) ± standard deviation (SD). The normal distribution of data was checked using Descriptive Statistics tests in the statistical package SPSS for Windows (v15.0, SPSS Inc.)**.** In order to evaluate the effects of the different treatment, the data were analysed by multivariate analysis of variance (ANOVA) and Tukey’s honestly significant difference (HSD) procedure, as well as post hoc tests. A linear Pearson’s correlation was used to quantify the strength (0.1–0.3 weak, 0.3–0.6 moderate, 0.7–0.9 strong) of the relationship between the variables [56]. The correlation coefficients were calculated using the statistical package SPSS for Windows (v15.0, SPSS Inc., Chicago, IL, USA). The results were recognised as statistically significant at *p* ≤ 0.05.

## Figures and Tables

**Table 1:** Acidity, microbiological parameters, and sugar concentration in processed wheat bran.

Samples	pH		TTA, °N		Lactic Acid Content, g/100 g		LAB	M/Y	TBC	TEC	Fructose	Glucose	Sucrose	Maltose
	Duration of Fermentation, h				L(+)	D(−)	log_10_ CFU/g				g/100 g			
	0	24	0	24										
**W_Con_**	6.04 ± 0.01b	−	0.10 ± 0.02a	−	−	−	5.20 ± 0.12c	4.26 ± 0.11b	9.04 ± 0.14c	5.69 ± 0.23c	0.16 ± 0.04ab	0.55 ± 0.06a	nd	0.31 ± 0.03c
**W_ConLpl_**		3.49 ± 0.02a		4.20 ± 0.20c	0.065 ± 0.003a	1.988 ± 0.015d	8.50 ± 0.14d	3.64 ± 0.10a	9.55 ± 0.17d	nd	0.19 ± 0.03b	0.69 ± 0.02b	nd	0.35 ± 0.02c
**W_ConLu_**		3.46 ± 0.01a		4.30 ± 0.20c	0.070 ± 0.004a	1.868 ± 0.024d	9.09 ± 0.15e	4.85 ± 0.12d	9.55 ± 0.16d	nd	0.12 ± 0.01a	0.75 ± 0.04c	nd	0.32 ± 0.03c
**W_ex115_**	6.17 ± 0.02c	−	0.10 ± 0.03a	−	−	−	4.85 ± 0.09b	4.20 ± 0.12b	8.93 ± 0.10c	4.92 ± 0.14b	nd	nd	0.17 ± 0.02a	0.48 ± 0.04d
**W_ex115/Lpl_**		3.81 ± 0.02b		5.50 ± 0.20e	0.666 ± 0.011d	0.899 ± 0.016c	8.69 ± 0.13d	4.18 ± 0.10b	8.92 ± 0.12c	nd	0.18 ± 0.01b	nd	nd	0.15 ± 0.01b
**W_ex115/Lu_**		3.91 ± 0.01b		4.80 ± 0.10d	0.237 ± 0.007b	0.770 ± 0.012b	8.65 ± 0.11d	4.25 ± 0.09b	8.90 ± 0.11c	nd	0.11 ± 0.01a	nd	nd	0.39 ± 0.02c
**W_ex130/16_**	6.13 ± 0.02c	−	0.10 ± 0.02a	−	−	−	3.17 ± 0.09a	4.37 ± 0.10b	8.70 ± 0.19b	4.67 ± 0.16b	nd	nd	0.42 ± 0.05c	0.16 ± 0.02b
**W_ex130/16/Lpl_**		4.02 ± 0.01b		3.80 ± 0.10b	0.524 ± 0.010d	0.683 ± 0.009b	8.43 ± 0.14d	4.48 ± 0.08c	8.65 ± 0.11b	nd	0.12 ± 0.01a	nd	nd	nd
**W_ex130/16/Lu_**		4.28 ± 0.01c		3.30 ± 0.10a	0.438 ± 0.003c	0.317 ± 0.014a	8.54 ± 0.09d	4.39 ± 0.12b	8.79 ± 0.12b	nd	nd	nd	0.14 ± 0.01a	nd
**W_ex130/20_**	5.96 ± 0.01a	−	0.20 ± 0.04b	−	−	−	4.41 ± 0.11b	4.20 ± 0.12b	8.65 ± 0.11b	4.79 ± 0.15b	nd	nd	0.26 ± 0.03b	0.10 ± 0.02a
**W_ex130/20/Lpl_**		3.90 ± 0.01b		5.50 ± 0.20e	0.440 ± 0.014c	0.385 ± 0.017a	8.75 ± 0.11d	4.25 ± 0.10b	8.95 ± 0.13c	nd	0.12 ± 0.01a	nd	nd	nd
**W_ex130/20/Lu_**		4.02 ± 0.02b		4.90 ± 0.10d	0.449 ± 0.012c	0.360 ± 0.023a	8.62 ± 0.12d	4.17 ± 0.13b	8.74 ± 0.12b	nd	0.11 ± 0.01a	nd	nd	nd
**W_ex130/25_**	5.91 ± 0.02a	−	0.20 ± 0.03b	−	−	−	5.34 ± 0.09c	4.38 ± 0.19b	8.46 ± 0.10a	4.32 ± 0.14a	0.11 ± 0.02a	nd	0.81 ± 0.07d	0.11 ± 0.01a
**W_ex130/25/Lpl_**		4.22 ± 0.02c		3.90 ± 0.10b	0.423 ± 0.009c	0.322 ± 0.014a	8.46 ± 0.11d	4.29 ± 0.07b	8.63 ± 0.12ab	nd	nd	nd	nd	nd
**W_ex130/25/Lu_**		4.20 ± 0.01c		3.50 ± 0.10a	0.275 ± 0.013b	0.203 ± 0.007a	8.79 ± 0.12d	4.32 ± 0.07b	8.84 ± 0.13b	nd	nd	nd	nd	nd

W—wheat bran; Con—non-extruded control; Lpl—fermented with *Lactobacillus plantarum*; Lu—fermented with *L. uvarum*; _ex115_—extruded at 115 °C with a screw speed of 16 rpm; _ex130/screwspeed16_—extruded at 130 °C and 16 rpm; _ex130/screwspeed20_—extruded at 130 °C and 20 rpm; _ex130/screwspeed25_—extruded at 130 °C and 25 rpm; TTA—total titratable acidity; LAB—lactic acid bacteria; M/Y—mould and yeast count; TBC—total bacteria count; TEC—total enterobacteria count; CFU—colony-forming units; nd—not detected; −—not analysed. Data expressed as mean values (*n* = 5) ± standard deviation (SD). a–e—means within a lines with different letters are significantly different (*p* ≤ 0.05).

**Table 2:** Amino acid concentration (g/100 g) in processed wheat bran.

Samples	Asp	Glu	Asn	Ser	His	Gly	Thr	Arg	Ala	Tyr	Cys	Val	Met	Trp	Phe	Ile	Leu	Lys	Pro
**W_Con_**	0.43 ± 0.03a	1.75 ± 0.09b	nd	0.29 ± 0.03a	0.12 ± 0.01a	0.27 ± 0.02a	0.25 ± 0.02a	0.31 ± 0.03a	0.24 ± 0.02a	0.18 ± 0.01a	0.34 ± 0.03a	0.34 ± 0.03a	0.12 ± 0.01a	0.36 ± 0.03b	0.28 ± 0.02ab	0.40 ± 0.04b	0.14 ± 0.01ab	0.26 ± 0.02a	0.50 ± 0.04d
**W_ConLpl_**	0.50 ± 0.04ab	1.91 ± 0.12b	nd	0.33 ± 0.02a	0.13 ± 0.01a	0.33 ± 0.03ab	0.30 ± 0.03a	0.37 ± 0.03b	0.29 ± 0.02b	0.19 ± 0.01a	0.38 ± 0.03a	0.43 ± 0.03b	0.10 ± 0.01a	0.36 ± 0.03b	0.24 ± 0.02a	0.45 ± 0.04b	0.15 ± 0.01b	0.27 ± 0.02a	1.17 ± 0.12f
**W_ConLu_**	0.48 ± 0.03a	2.05 ± 0.14bc	nd	0.34 ± 0.03a	0.16 ± 0.01a	0.32 ± 0.03ab	0.30 ± 0.03a	0.37 ± 0.03b	0.28 ± 0.02b	0.21 ± 0.02a	0.44 ± 0.04b	0.43 ± 0.04b	0.12 ± 0.01a	0.39 ± 0.03b	0.25 ± 0.02a	0.48 ± 0.04b	0.15 ± 0.01b	0.31 ± 0.03a	1.09 ± 0.07f
**W_ex115_**	0.44 ± 0.02a	1.92 ± 0.10b	nd	0.33 ± 0.03a	0.13 ± 0.01a	0.30 ± 0.03a	0.29 ± 0.02a	0.35 ± 0.03b	0.31 ± 0.03b	0.21 ± 0.02a	0.40 ± 0.03b	0.42 ± 0.04ab	0.10 ± 0.01a	0.37 ± 0.03b	0.23 ± 0.02a	0.46 ± 0.04b	0.15 ± 0.01b	0.30 ± 0.02a	0.82 ± 0.07e
**W_ex115/Lpl_**	0.45 ± 0.03a	1.89 ± 0.11b	nd	0.32 ± 0.02a	0.14 ± 0.01a	0.30 ± 0.03a	0.30 ± 0.03ab	0.33 ± 0.03ab	0.26 ± 0.02b	0.19 ± 0.01a	0.35 ± 0.03a	0.36 ± 0.03a	0.11 ± 0.01a	0.32 ± 0.03ab	0.22 ± 0.02a	0.42 ± 0.04b	0.13 ± 0.01a	0.29 ± 0.02a	0.80 ± 0.06e
**W_ex115/Lu_**	0.53 ± 0.04a	2.13 ± 0.08c	nd	0.37 ± 0.03b	0.15 ± 0.01a	0.36 ± 0.03b	0.35 ± 0.03b	0.39 ± 0.03b	0.37 ± 0.03b	0.21 ± 0.02a	0.43 ± 0.04b	0.46 ± 0.04b	0.10 ± 0.01a	0.40 ± 0.03b	0.26 ± 0.02a	0.50 ± 0.05b	0.17 ± 0.01b	0.38 ± 0.03b	1.03 ± 0.08f
**W_ex130/16_**	0.42 ± 0.03a	1.74 ± 0.14b	nd	0.32 ± 0.03a	0.11 ± 0.01a	0.29 ± 0.02a	0.29 ± 0.02a	0.33 ± 0.03ab	0.26 ± 0.02b	0.21 ± 0.02a	0.36 ± 0.03a	0.35 ± 0.03a	0.17 ± 0.01b	0.32 ± 0.03ab	0.22 ± 0.02a	0.43 ± 0.04b	0.14 ± 0.01ab	0.29 ± 0.02a	1.05 ± 0.07f
**W_ex130/16/Lpl_**	0.47 ± 0.03a	1.74 ± 0.13b	nd	0.31 ± 0.03a	0.10 ± 0.01a	0.32 ± 0.03ab	0.29 ± 0.02a	0.32 ± 0.03ab	0.33 ± 0.03b	0.20 ± 0.01a	0.36 ± 0.03a	0.35 ± 0.03a	0.18 ± 0.01b	0.33 ± 0.03a	0.22 ± 0.02a	0.42 ± 0.04b	0.15 ± 0.01b	0.29 ± 0.02a	1.08 ± 0.09f
**W_ex130/16/Lu_**	0.53 ± 0.04a	1.97 ± 0.15b	nd	0.32 ± 0.03a	0.11 ± 0.01a	0.30 ± 0.03a	0.30 ± 0.03ab	0.35 ± 0.03b	0.27 ± 0.02b	0.20 ± 0.01a	0.44 ± 0.03b	0.40 ± 0.03ab	0.17 ± 0.01b	0.42 ± 0.04b	0.32 ± 0.03b	0.44 ± 0.04b	0.14 ± 0.01ab	0.28 ± 0.02a	0.36 ± 0.03c
**W_ex130/20_**	0.45 ± 0.04a	1.48 ± 0.10a	nd	0.26 ± 0.02a	0.14 ± 0.01a	0.24 ± 0.02a	0.27 ± 0.02a	0.28 ± 0.02a	0.22 ± 0.02a	0.18 ± 0.01a	0.40 ± 0.03b	0.34 ± 0.03a	0.11 ± 0.01a	0.33 ± 0.03ab	0.24 ± 0.02a	0.34 ± 0.03a	0.12 ± 0.01a	0.34 ± 0.02b	0.29 ± 0.02b
**W_ex130/20/Lpl_**	0.50 ± 0.04a	1.49 ± 0.11a	nd	0.26 ± 0.02a	0.12 ± 0.01a	0.28 ± 0.02a	0.28 ± 0.02a	0.29 ± 0.02a	0.25 ± 0.02a	0.17 ± 0.01a	0.45 ± 0.04b	0.36 ± 0.03a	0.12 ± 0.01a	0.33 ± 0.03ab	0.24 ± 0.02a	0.34 ± 0.03a	0.12 ± 0.01a	0.35 ± 0.03b	0.18 ± 0.01a
**W_ex130/20/Lu_**	0.48 ± 0.02a	1.55 ± 0.12a	nd	0.27 ± 0.02a	0.12 ± 0.01a	0.27 ± 0.02a	0.28 ± 0.02a	0.29 ± 0.02a	0.24 ± 0.02a	0.18 ± 0.01a	0.42 ± 0.04b	0.36 ± 0.03a	0.11 ± 0.01a	0.33 ± 0.03ab	0.24 ± 0.02a	0.33 ± 0.03a	0.11 ± 0.01a	0.37 ± 0.03b	0.24 ± 0.02b
**W_ex130/25_**	0.44 ± 0.03a	1.43 ± 0.09a	nd	0.26 ± 0.02a	0.10 ± 0.01a	0.24 ± 0.02a	0.25 ± 0.02a	0.27 ± 0.02a	0.21 ± 0.02a	0.19 ± 0.01a	0.38 ± 0.03a	0.32 ± 0.03a	0.13 ± 0.01a	0.32 ± 0.03ab	0.24 ± 0.02a	0.32 ± 0.03a	0.10 ± 0.01a	0.29 ± 0.02a	0.27 ± 0.02b
**W_ex130/25/Lpl_**	0.48 ± 0.03a	1.48 ± 0.07a	nd	0.25 ± 0.02a	0.10 ± 0.01a	0.26 ± 0.02a	0.28 ± 0.02a	0.28 ± 0.02a	0.23 ± 0.02a	0.18 ± 0.01a	0.35 ± 0.03a	0.34 ± 0.03a	0.09 ± 0.01a	0.29 ± 0.02a	0.22 ± 0.02a	0.32 ± 0.03a	0.11 ± 0.01a	0.35 ± 0.03b	0.14 ± 0.01a
**W_ex130/25/Lu_**	0.48 ± 0.04a	1.47 ± 0.08a	nd	0.26 ± 0.02a	0.11 ± 0.01a	0.26 ± 0.02a	0.26 ± 0.02a	0.27 ± 0.02a	0.23 ± 0.02a	0.17 ± 0.01a	0.40 ± 0.03ab	0.34 ± 0.03a	0.13 ± 0.01a	0.29 ± 0.02a	0.22 ± 0.02a	0.32 ± 0.03a	0.11 ± 0.01a	0.34 ± 0.03b	0.24 ± 0.02b

W—wheat bran; Con—non-extruded wheat bran; Lpl—fermented with *Lactobacillus plantarum*; Lu—fermented with *L. uvarum*; _ex115_—extruded at 115 °C with a screw speed of 16 rpm; _ex130/screwspeed16_—extruded at 130 °C and 16 rpm; _ex130/screwspeed20_—extruded at 130 °C and 20 rpm; _ex130/screwspeed25_—extruded at 130 °C and 25 rpm; nd—not detected; Asp—aspartic acid; Ala—alanine; Gly—glycine; Val—valine; Leu—leucine; Ile—isoleucine; Thr—threonine; Ser—serine; Pro—proline; Asn—asparagine; Met—methionine; Glu—glutamine; Phe—phenylalanine; Lys—lysine; His—histidine; Arg—arginine; Tyr—tyrosine; Trp—tryptophan; Cys—cysteine. Data expressed as mean values (*n* = 5) ± standard deviation (SD). a–f—means within a lines with different letters are significantly different (*p* ≤ 0.05).

**Table 3:** Biogenic amines concentration (mg/kg) in processed wheat bran.

Samples	PUT	CAD	HIST	SPRM	TYR	PHE	SPRMD
**W_Con_**	91.3 ± 2.4a	33.8 ± 2.0a	9.2 ± 0.4a	35.9 ± 2.5b	nd	nd	nd
**W_ConLpl_**	88.6 ± 3.7a	48.9 ± 3.5b	nd	31.8 ± 1.7b	nd	nd	nd
**W_ConLu_**	109.3 ± 5.2b	nd	nd	26.2 ± 0.9a	nd	nd	nd
**W_ex115_**	105.1 ± 4.3b	nd	nd	35.2 ± 2.6b	nd	nd	nd
**W_ex115/Lpl_**	138.6 ± 5.4e	nd	nd	44.2 ± 3.4c	nd	nd	nd
**W_ex115/Lu_**	139.6 ± 6.1e	nd	nd	34.9 ± 2.5b	nd	nd	nd
**W_ex130/16_**	134.2 ± 3.4e	nd	nd	30.6 ± 2.1b	nd	nd	nd
**W_ex130/16/Lpl_**	115.4 ± 5.4bc	nd	nd	33.9 ± 1.9b	nd	nd	nd
**W_ex130/16/Lu_**	122.1 ± 2.8cd	nd	nd	31.6 ± 2.0b	nd	nd	nd
**W_ex130/20_**	150.6 ± 6.3f	nd	nd	31.5 ± 0.7b	nd	nd	nd
**W_ex130/20/Lpl_**	126.6 ± 2.9d	nd	nd	31.2 ± 1.8b	nd	nd	nd
**W_ex130/20/Lu_**	154.1 ± 4.7f	nd	nd	32.5 ± 2.3b	nd	nd	nd
**W_ex130/25_**	107.0 ± 5.4b	nd	nd	25.3 ± 2.1a	nd	nd	nd
**W_ex130/25/Lpl_**	160.1 ± 7.2f	nd	nd	33.6 ± 3.1b	nd	nd	nd
**W_ex130/25/Lu_**	91.3 ± 2.1a	33.8 ± 2.1a	9.2 ± 0.3a	35.9 ± 2.7b	nd	nd	nd

W—wheat bran; Con—non-extruded control; Lpl—fermented with *Lactobacillus plantarum*; Lu—fermented with *L. uvarum*; _ex115_—extruded at 115 °C with a screw speed of 16 rpm; _ex130/16_—extruded at 130 °C and 16 rpm; _ex130/20_—extruded at 130 °C and 20 rpm; _ex130/25_—extruded at 130 °C and 25 rpm; PUT—putrescine; CAD—cadaverine; HIST—histamine; SPRM—spermine; PHE—phenylethylamine; TYR—tyramine; SPRMD—spermidine; nd—not detected. Data are represented as means (*n* = 5) ± SE. a–f—mean values within a lines denoted with different letters are significantly different (*p* ≤ 0.05).

**Table 4:** Mycotoxin concentration (µg/kg) in processed wheat bran.

Samples	AOH	AME	17-DMAG	15-DON	DON	D3G	15ACS	ENN A	ENN A1	FB1	FB2	MEL	STC	OTB	OTA	T-2	HT-2	FUSX	Neo	AFB1
**W_Con_**	1.76 ± 0.13d	nd	nd	50.18 ± 1.21e	58.8 ± 0.69f	3.93 ± 0.29h	15.28± 1.17d	5.31 ± 0.27c	1.24 ± 0.08c	nd	nd	0.3 ± 0.02d	nd	nd	7.66 ± 0.37d	1.81 ± 0.15c	3.76 ± 0.38f	7.66 ± 0.37d	nd	3.2 ± 0.21d
**W_ConLpl_**	0.87 ± 0.07a	nd	nd	36.77 ± 0.25c	26.5 ± 0.17c	0.61 ± 0.05c	21.99 ± 0.21e	2.29 ± 0.14b	0.44 ± 0.03b	nd	nd	0.19 ± 0.01bc	nd	nd	nd	1.7 ± 0.15c	0.27 ± 0.09a	nd	nd	nd
**W_ConLu_**	0.72 ± 0.04a	nd	nd	41.73 ± 0.36d	27 ± 0.12c	0.8 ± 0.04d	19.2 ± 0.12d	1.75 ± 0.09a	0.41 ± 0.03b	nd	nd	0.22 ± 0.02c	0.09 ± 0.01a	nd	nd	nd	0.2 ± 0.06a	nd	nd	nd
**W_ex115_**	0.85 ±0.07a	nd	nd	75.17 ± 0.63g	45.1 ± 0.58e	2.79 ± 0.17g	1.2 ± 0.11a	1.61 ± 0.12a	0.35 ± 0.02b	nd	nd	0.43 ± 0.03e	0.11 ± 0.01a	nd	1.68 ± 0.13c	0.89 ± 0.04b	2.85 ± 0.11e	4.05 ± 0.21b	0.1 ± 0.01b	2.55 ± 0.19c
**W_ex115/Lpl_**	0.9 ± 0.04a	1.09 ± 0.09c	nd	63.36 ± 0.29f	45.8 ± 0.27e	2.68 ± 0.12g	1.68 ± 0.07b	39.51 ± 0.05f	9.65 ± 0.11g	nd	nd	0.28 ± 0.02d	0.33 ± 0.02b	0.08 ± 0.01a	2.92 ± 0.14d	2.56 ± 0.13d	0.61 ± 0.05b	nd	0.05 ±0.01a	nd
**W_ex115/Lu_**	0.81 ± 0.03a	0.88 ± 0.06b	nd	51.09 ± 0.34e	53 ± 0.32f	1.92 ± 0.09f	1.8 ± 0.09b	25.53 ± 0.07e	6.22 ± 0.09f	nd	nd	0.16 ± 0.01b	0.29 ± 0.02b	0.07 ± 0.01a	nd	nd	1 ± 0.08d	nd	0.06 ± 0.01a	nd
**W_ex130/16_**	1 ± 0.05b	0.85 ± 0.04b	0.34 ± 0.01a	106.45 ± 2.67i	32.9 ± 0.26d	1.83 ± 0.12f	nd	1.34 ± 0.28a	0.25 ± 0.02a	nd	nd	0.19 ± 0.01b	0.7 ± 0.06c	nd	0.63 ± 0.03b	0.98 ± 0.06b	nd	4.54 ± 0.27b	0.08 ± 0.01ab	1.7 ± 0.09b
**W_ex130/16/Lpl_**	0.88 ± 0.04a	nd	0.37 ± 0.03a	24.24 ± 0.11b	30.8 ± 0.19d	1.13 ± 0.06e	1.71 ± 0.14b	11.48 ± 0.22d	2.52 ± 0.11e	nd	nd	0.07 ± 0.01a	0.96 ± 0.05d	nd	nd	2.41 ± 0.14d	0.63 ± 0.05b	nd	nd	nd
**W_ex130/16/Lu_**	0.95 ± 0.05ab	nd	0.39 ± 0.02a	nd	30.2 ± 0.15d	0.98 ± 0.05d	1.66 ± 0.11b	10.18 ± 0.09d	1.87 ± 0.06d	nd	nd	0.06 ± 0.01a	0.75 ± 0.04c	nd	6.44 ± 0.21e	nd	0.86 ± 0.06c	nd	0.04 ±0.01a	nd
**W_ex130/20_**	1.8 ± 0.12d	0.51 ± 0.03a	0.79 ± 0.04b	85.65 ± 1.17h	24.4 ± 0.25c	1.05 ± 0.09d	1.31 ± 0.11a	1.29 ± 0.14a	0.25 ± 0.02a	0.91 ± 0.07d	0.07 ± 0.01	0.16 ± 0.02b	1.76 ± 0.08g	nd	0.29 ± 0.02a	0.85 ± 0.05b	nd	4.9 ± 0.15c	0.06 ± 0.01a	0.91 ± 0.04a
**W_ex130/20/Lpl_**	0.86 ± 0.05a	nd	0.86 ± 0.06b	20.93 ± 0.12a	21.1 ± 0.09b	0.26 ± 0.02a	2.18 ± 0.10c	2.17 ± 0.09b	0.42 ± 0.02b	0.56 ± 0.05c	nd	0.03 ± 0.01a	2.11 ± 0.12h	nd	nd	nd	1.02 ± 0.07d	nd	0.04 ± 0.01a	nd
**W_ex130/20/Lu_**	3.42 ± 0.23e	0.63 ± 0.04a	1.12 ± 0.09c	nd	24.1 ± 0.10c	0.3 ± 0.02a	1.43 ± 0.12a	2.21 ± 0.10b	0.36 ± 0.03b	0.41 ± 0.04b	nd	nd	1.75 ± 0.16g	nd	nd	nd	0.85 ± 0.05c	3.52 ± 0.14a	0.03 ± 0.01a	nd
**W_ex130/25_**	3.29 ± 0.17e	3.45 ± 0.15e	0.82 ± 0.04b	83.45 ± 1.27h	23.2 ± 0.13c	1.11 ± 0.07e	nd	1.29 ± 0.09a	0.26 ± 0.02a	0.07 ±0.01a	nd	0.05± 0.01a	1.96± 0.14gh	nd	0.54 ± 0.03b	1.48 ± 0.09c	nd	4.35 ± 0.17b	0.07 ± 0.01a	0.92 ± 0.04a
**W_ex130/25/Lpl_**	1.37 ± 0.10c	1.01 ± 0.08c	1.05 ± 0.07c	18.82 ± 0.10a	19.5 ± 0.12a	0.51 ± 0.04b	1.44 ± 0.13a	1.58 ± 0.17a	0.31 ± 0.02b	0.04 ± 0.01a	nd	0.04 ± 0.01a	1.43 ± 0.11f	nd	nd	nd	1.05 ± 0.04d	nd	0.05 ± 0.01a	nd
**W_ex130/25/Lu_**	1.31 ± 0.12c	1.4 ± 0.09d	0.78 ± 0.06b	nd	19.9 ± 0.14a	0.44 ± 0.02b	1.81 ± 0.15b	1.29 ± 0.09a	0.26 ± 0.02a	0.08 ± 0.01a	nd	0.02 ± 0.01a	1.27 ± 0.09e	nd	nd	nd	1.17 ± 0.06d	nd	0.05 ± 0.02a	nd

W—wheat bran; Con—non-extruded control; Lpl—fermented with *Lactobacillus plantarum*; Lu—fermented with *L. uvarum*; _ex115_—extruded at 115 °C with a screw speed of 16 rpm; _ex130/screwspeed16_—extruded at 130 °C and 16 rpm; _ex130/screwspeed20_—extruded at 130 °C and 20 rpm; _ex130/screwspeed25_—extruded at 130 °C and 25 rpm; AOH—alternariol; AME—alternariol monomethyl ether; 17-DMAG—17-dimethylaminoethylamino-17-demethoxygeldanamycin; 15-DON—15-acetyldeoxynivalenol; MEL—meleagrin; Neo—neosolaniol; 15ACS—15-acetoxyscirpenol; ENN A—enniatin A; ENN A1—enniatin A1; FB1—fumonisin B1; FB2—fumonisin B2; DON—deoxynivalenol; STC—sterigmatocystin; OTB—ochratoxin B; FUSX—fusarenon X; T-2—T-2 toxin; HT-2—HT-2 toxin; OTA—ochratoxin A; D3G—deoxynivalenol-3-glucoside; AFB1—aflatoxin B1; nd—not detected. Data are presented as means (*n* = 5) ± standard deviation (SD). a–i—means within a lines with different letters are significantly different (*p* ≤ 0.05).

## Data Availability

Data available in a publicly accessible repository. The data presented in this study are openly available in (repository name e.g., FigShare) at (doi), reference number.

## Supplementary Materials

The following are available online at https://www.mdpi.com/2072-6651/13/2/163/s1, Supplementary Material S1. Correlation coefficients, Table S1: Influence of fermentation, extrusion and their interaction on analysed parameters of wheat bran.
