# Supplementary material for: Combination of Extrusion and Fermentation with Lactobacillus plantarum and L. uvarum Strains for Improving the Safety Characteristics of Wheat Bran
Source: Toxins (Basel). 2021 Feb 19;13(2):163. doi: 10.3390/toxins13020163 (PMC7923204; doi:10.3390/toxins13020163)
Supplement: Supplementary file 1 [file toxins-13-00163-s001.pdf]

Supplementary Materials: Combination of Extrusion and Fermentation with *Lactobacillus plantarum* and *L. uvarum* Strains for Improving the Safety Characteristics of Wheat Bran

Elena Bartkiene, Egle Zokaityte, Vita Lele, Vytaute Starkute, Paulina Zavistanaviciute, Dovile Klupsaite, Darius Cernauskas, Modestas Ruzauskas, Vadims Bartkevics, Iveta Pugajeva, Zane Bērziņa, Romas Gruzauskas, Sonata Sidlauskiene, Antonello Santini and Grazina Juodeikiene

Supplementary Material S1. Correlation coefficients.

		pH	TTA
pH	Pearson Correlation	1	-0.957**
	Sig. (1-tailed)		0.0001
	N	45	45
TTA	Pearson Correlation	-0.957**	1
	Sig. (1-tailed)	0.0001	
	N	45	45

**. Correlation is significant at the 0.01 level (1-tailed).

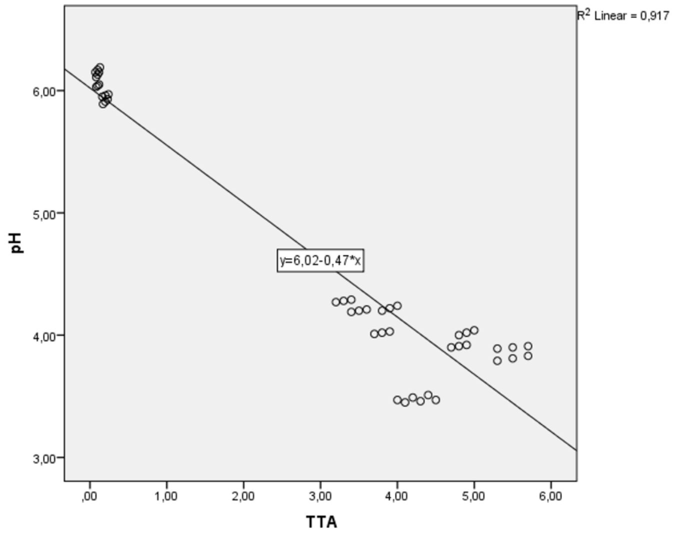

pH	Pearson Correlation	1	0.556**
	Sig. (1-tailed)		0.001
	N	30	30
L	Pearson Correlation	0.556**	1
	Sig. (1-tailed)	0.001	
	N	30	30
**. Correlation is significant at the 0.01 level (1-tailed).			

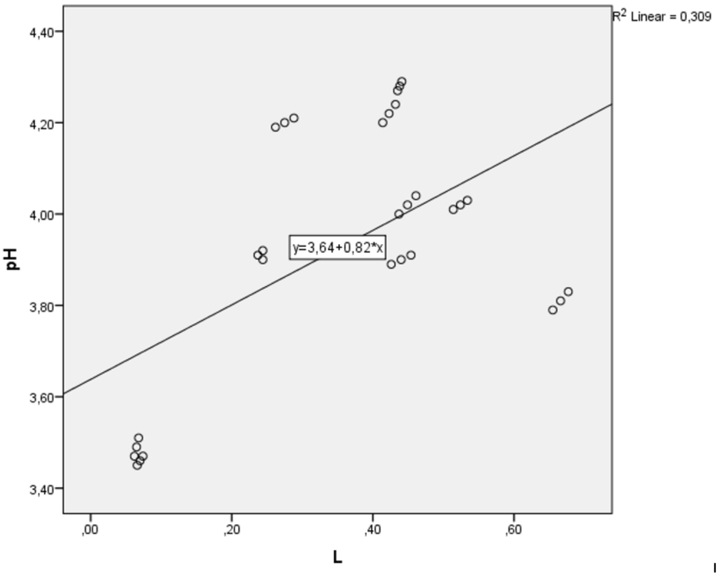

pH	Pearson Correlation	1	-0.932**
	Sig. (1-tailed)		0.0001
	N	30	30
D	Pearson Correlation	-0.932**	1
	Sig. (1-tailed)	0.0001	
	N	30	30
**. Correlation is significant at the 0.01 level (1-tailed).			

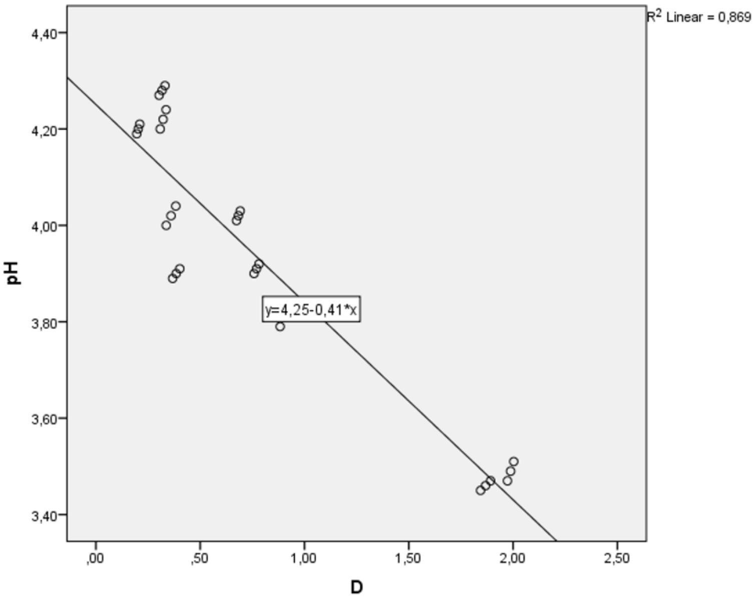

TTA	Pearson Correlation	1	0.273
	Sig. (2-tailed)		0.144
	N	30	30
L	Pearson Correlation	0.273	1
	Sig. (2-tailed)	0.144	
	N	30	30

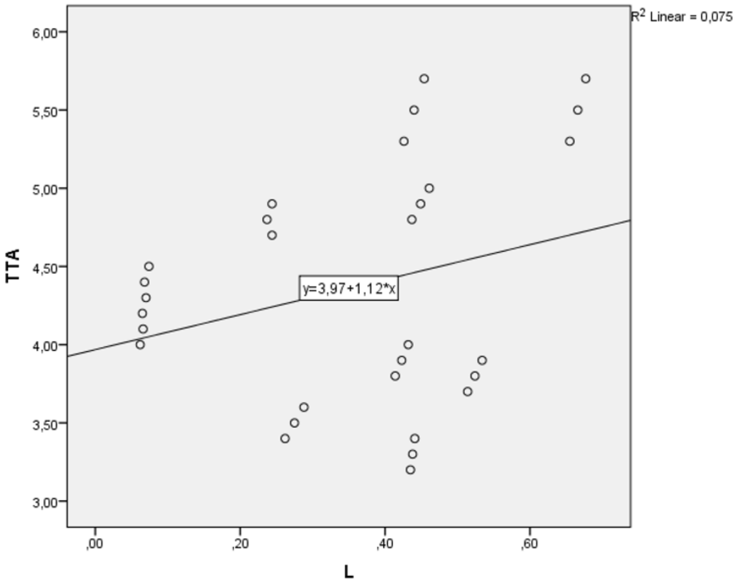

TTA	Pearson Correlation	1	.100
	Sig. (2-tailed)		.598
	N	30	30
D	Pearson Correlation	.100	1
	Sig. (2-tailed)	.598	
	N	30	30

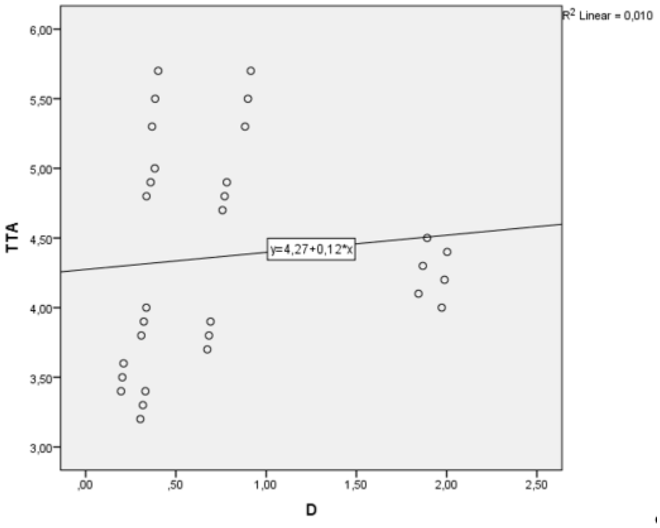

LAB	Pearson Correlation	1	-0.958**
	Sig. (2-tailed)		0.0001
	N	45	45
pH	Pearson Correlation	-0.958**	1
	Sig. (2-tailed)	0.0001	
	N	45	45
**. Correlation is significant at the 0.01 level (2-tailed).			

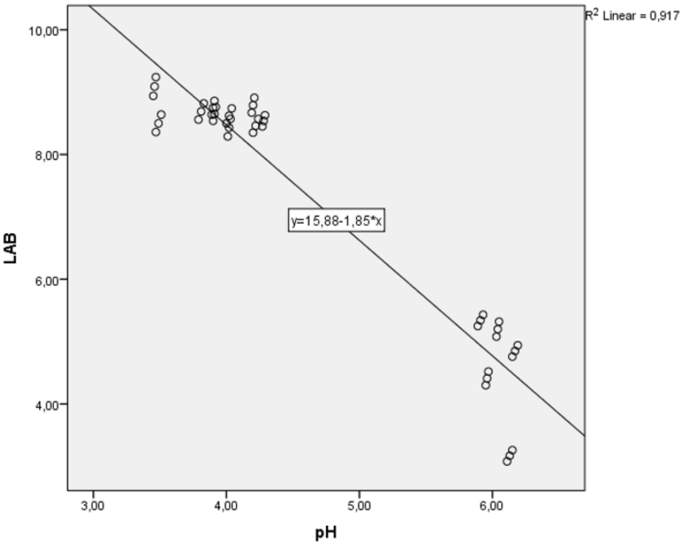

LAB	Pearson Correlation	1	0.935**
	Sig. (2-tailed)		0.0001
	N	45	45
TTA	Pearson Correlation	0.935**	1
	Sig. (2-tailed)	0.0001	
	N	45	45
**. Correlation is significant at the 0.01 level (2-tailed).			

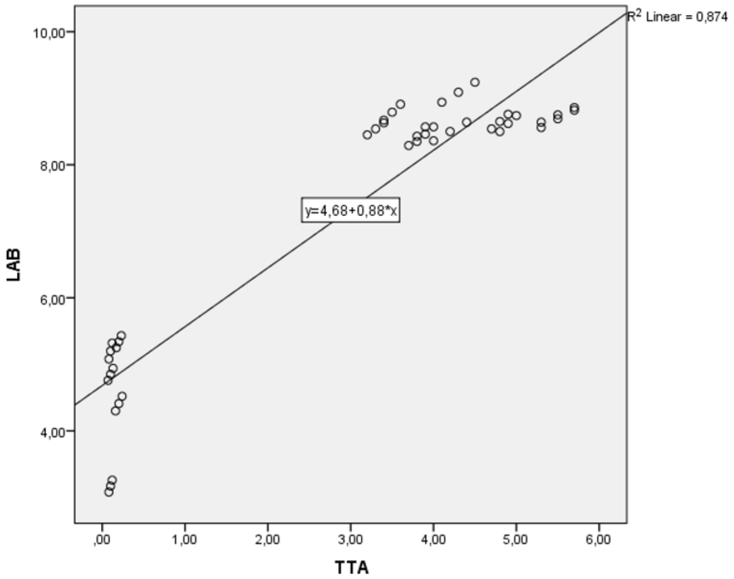

pH	Pearson Correlation	1	0.653**
	Sig. (2-tailed)		0.0001
	N	45	45
Sucrose	Pearson Correlation	0.653**	1
	Sig. (2-tailed)	0.0001	
	N	45	45
**. Correlation is significant at the 0.01 level (2-tailed).			

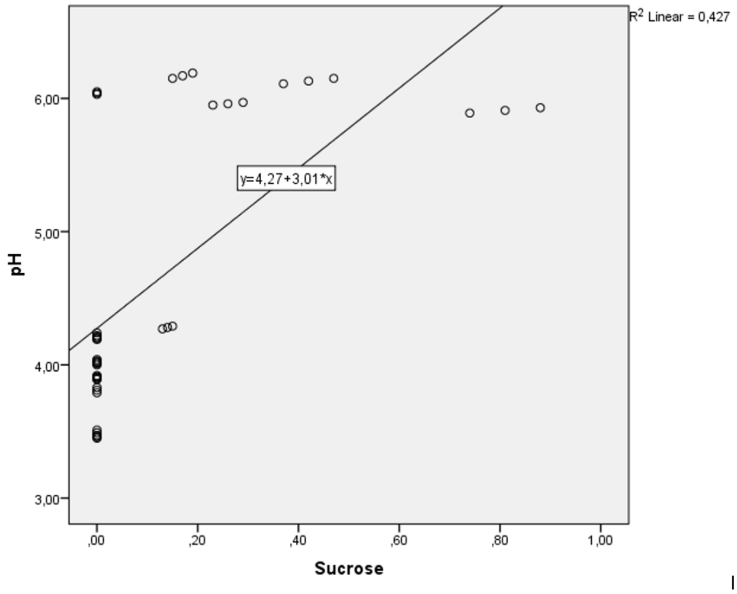

TTA	Pearson Correlation	1	-0.661**
	Sig. (2-tailed)		0.000
	N	45	45
Sucrose	Pearson Correlation	-0.661**	1
	Sig. (2-tailed)	0.0001	
	N	45	45
**. Correlation is significant at the 0.01 level (2-tailed).			

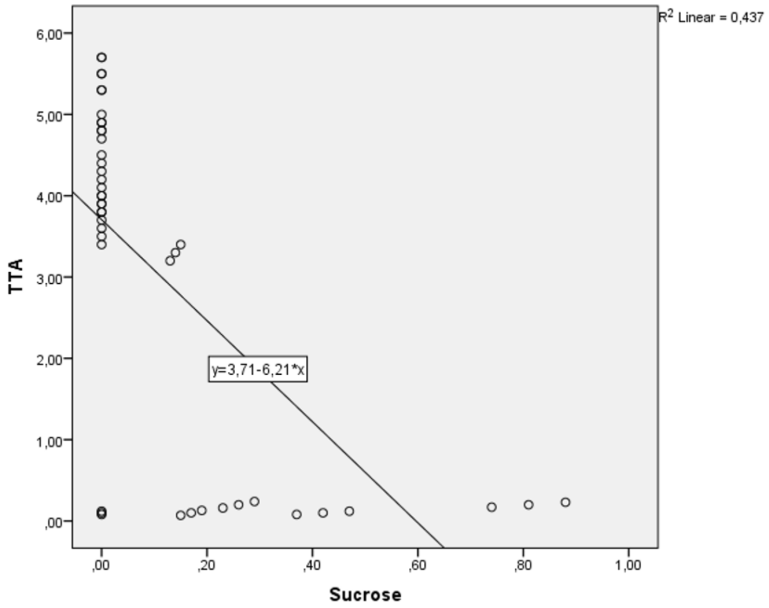

LAB	Pearson Correlation	1	-0.655**
	Sig. (2-tailed)		0.0001
	N	45	45
Sucrose	Pearson Correlation	-0.655**	1
	Sig. (2-tailed)	0.0001	
	N	45	45
**. Correlation is significant at the 0.01 level (2-tailed).			

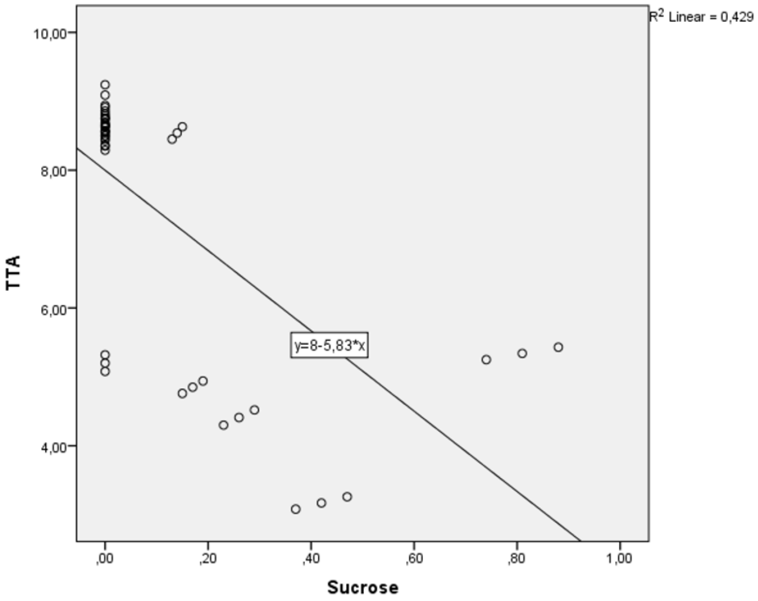

pH	Pearson Correlation	1	-0.411**
	Sig. (2-tailed)		0.005
	N	45	45
Fructose	Pearson Correlation	-0.411**	1
	Sig. (2-tailed)	0.005	
	N	45	45
**. Correlation is significant at the 0.01 level (2-tailed).			

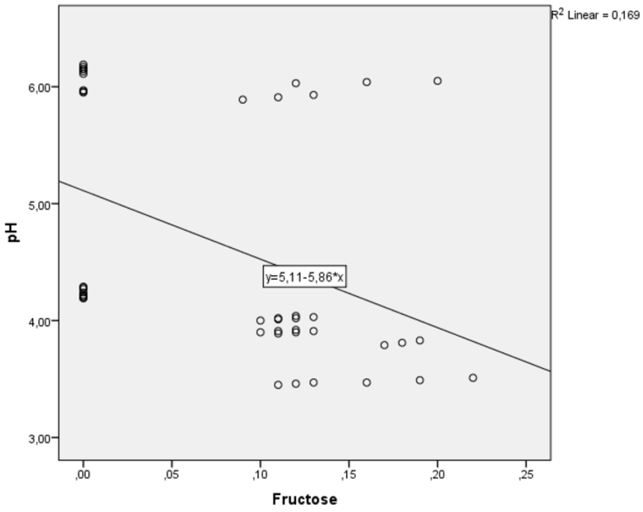

TTA	Pearson Correlation	1	0.413**
	Sig. (2-tailed)		0.005
	N	45	45
Fructose	Pearson Correlation	0.413**	1
	Sig. (2-tailed)	0.005	
	N	45	45
**. Correlation is significant at the 0.01 level (2-tailed).			

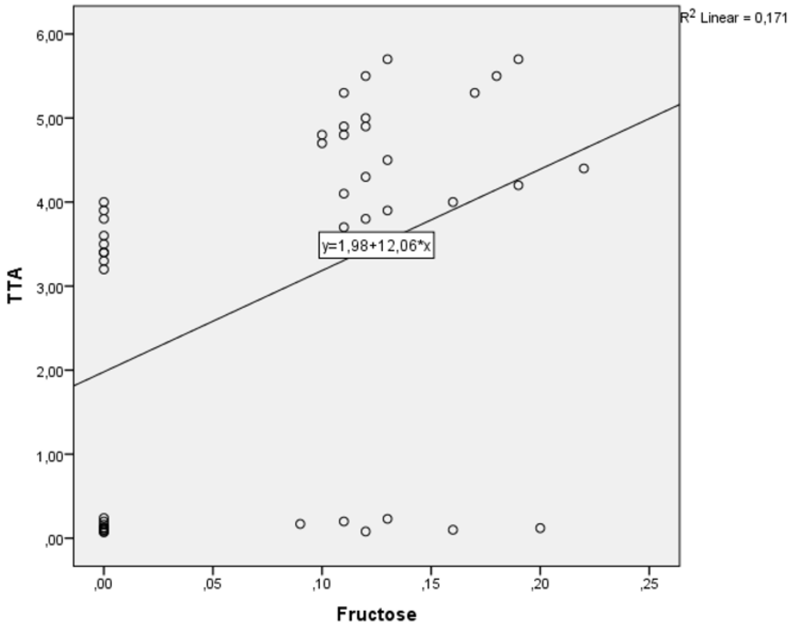

LAB	Pearson Correlation	1	0.358*
	Sig. (2-tailed)		0.016
	N	45	45
Fructose	Pearson Correlation	0.358*	1
	Sig. (2-tailed)	0.016	
	N	45	45
*. Correlation is significant at the 0.05 level (2-tailed).			

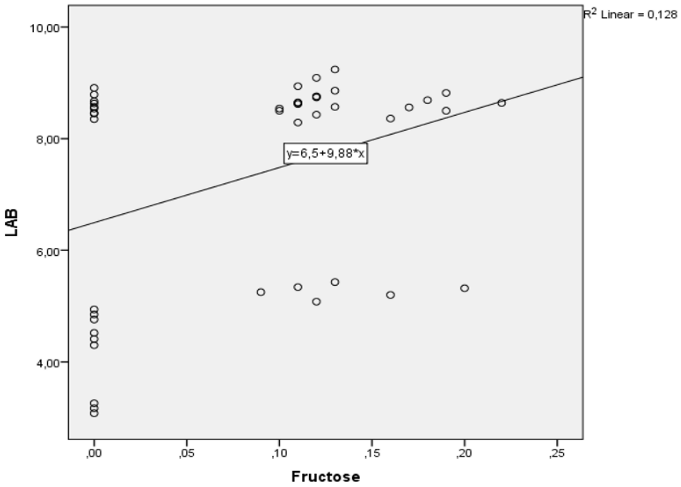

pH	Pearson Correlation	1	0.201
	Sig. (2-tailed)		0.186
	N	45	45
Maltose	Pearson Correlation	0.201	1
	Sig. (2-tailed)	0.186	
	N	45	45

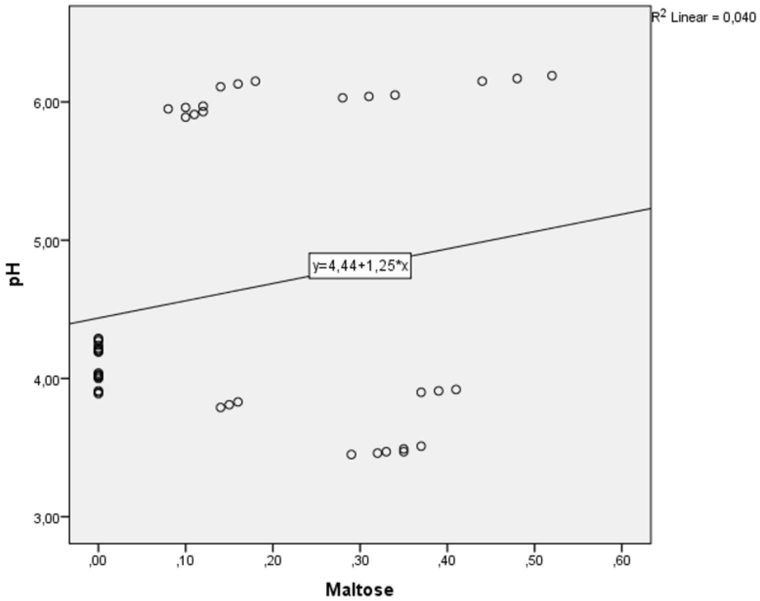

pH	Pearson Correlation	1	-0.218
	Sig. (2-tailed)		0.150
	N	45	45
Glucose	Pearson Correlation	-0.218	1
	Sig. (2-tailed)	0.150	
	N	45	45

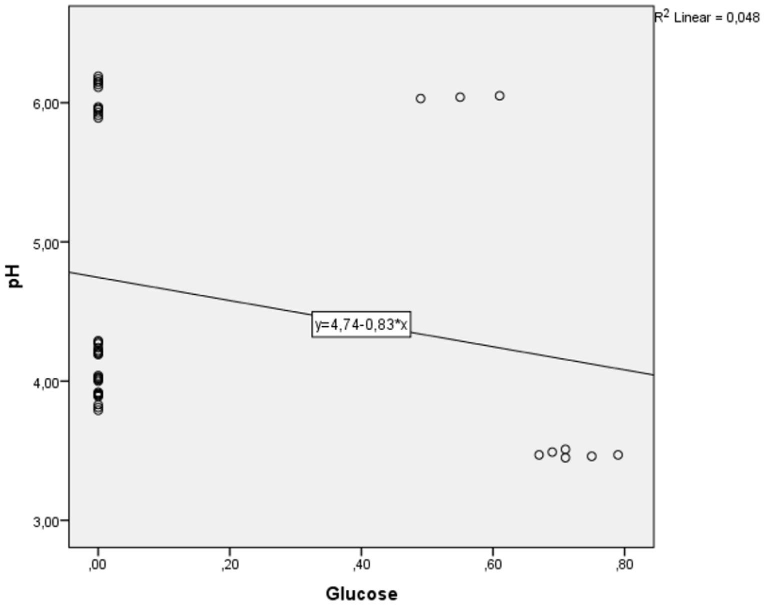

TTA	Pearson Correlation	1	-0.258
	Sig. (2-tailed)		0.087
	N	45	45
Maltose	Pearson Correlation	-0.258	1
	Sig. (2-tailed)	0.087	
	N	45	45

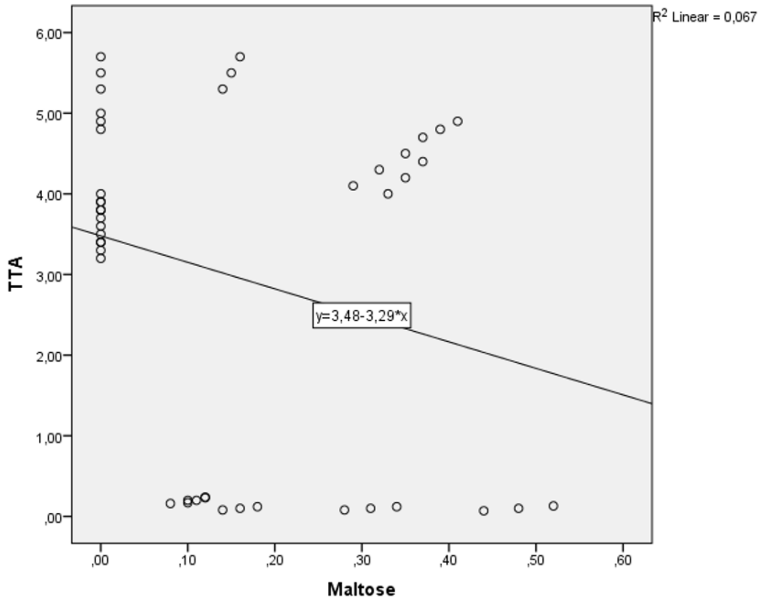

LAB	Pearson Correlation	1	-0.257
	Sig. (2-tailed)		0.088
	N	45	45
Maltose	Pearson Correlation	-0.257	1
	Sig. (2-tailed)	0.088	
	N	45	45

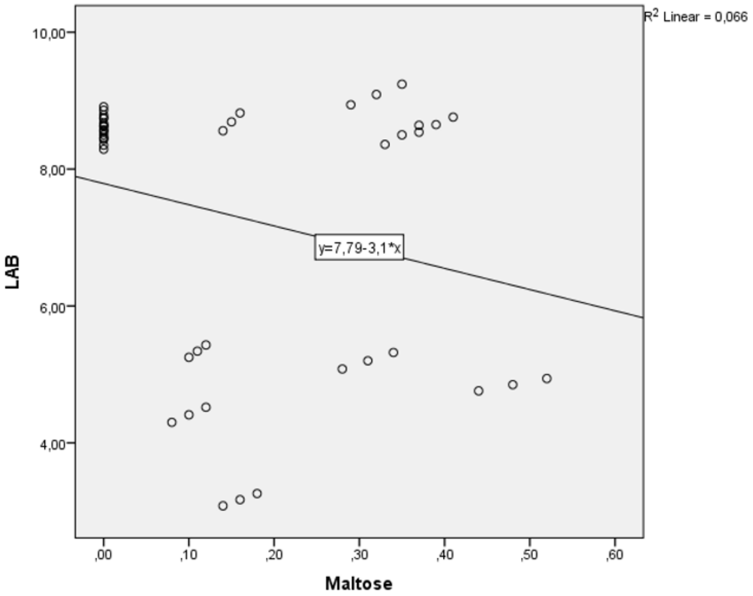

Table S1. Influence of fermentation, extrusion and their interaction on analysed parameters of wheat bran.

Tests of Between-Subjects Effects						
**Source**	**Dependent Variable**	**Type III Sum of Squares**	**df**	**Mean Square**	**F**	**Sig.**
LAB fermentation	pH	0.055	1	0.055	252.136	0.000
	Total titratable acidity (TTA)	1.323	1	1.323	60.136	0.000
	L(+) lactic acid	0.125	1	0.125	1446.010	0.000
	D(−) lactic acid	0.173	1	0.173	680.143	0.000
	LAB count	164.896	2	82.448	6104.227	0.000
	Mould/Yeast	0.390	2	0.195	15.637	0.000
	Total Bacteria Count	0.388	2	0.194	11.297	0.000
	Total Enterobacteria Count	237.949	2	118.974	12,729.075	0.000
	Fructose	0.039	2	0.019	82.886	0.000
	Glucose	0.013	2	0.006	16.929	0.000
	Sucrose	1.017	2	0.509	866.864	0.000
	Maltose	0.136	2	0.068	196.788	0.000
	Aspartic acid	0.032	2	0.016	14.443	0.000
	Glutamine	0.243	2	0.122	9.371	0.001
	Serine	0.004	2	0.002	2.874	0.072
	Histidine	0.001	2	0.001	6.200	0.006
	Glycine	0.010	2	0.005	8.179	0.001
	Threonine	0.006	2	0.003	5.506	0.009
	Arginine	0.005	2	0.003	3.686	0.037
	Alanine	0.008	2	0.004	7.560	0.002
	Tyrosine	0.001	2	0.000	1.778	0.186
	Cysteine	0.024	2	0.012	11.061	0.000
	Valine	0.015	2	0.008	7.288	0.003
	Methionine	0.000	2	0.000	1.800	0.183
	Tryptophan	0.012	2	0.006	7.023	0.003
	Phenylalanine	0.007	2	0.003	7.800	0.002
	Isoleucine	0.026	2	0.013	2.138	0.135
	Leucine	0.000	2	0.000	1.400	0.262
	Lysine	0.012	2	0.006	10.300	0.000
	Proline	0.073	2	0.036	11.077	0.000
	Mycotoxins					
	AOH	4,448	2	2,224	207,864	0.000
	AME	2,322	2	1,161	329,835	0.000
	17-DMAG	0.045	2	0.022	13,585	0.000
	15-DON	31,212,349	2	15,606,174	18,878,437	0.000
	MEL	0.148	2	0.074	327,265	0.000
	Neo	0.009	2	0.005	64,636	0.000
	15ACS	40,708	2	20,354	197,124	0.000
	ENN A	659,795	2	329,898	14,550,024	0.000
	ENN A1	36,884	2	18,442	5,787,282	0.000
	FB1	0.079	2	0.040	63,968	0.000
	FB2	0.002	2	0.001	147,000	0.000
	DON	535,756	2	267,878	3,117,761	0.000
	STC	0.139	2	0.070	11,014	0.000
	OTB	0.002	2	0.001	85,500	0.000

	FUSX	229,152	2	114,576	5,289,754	0.000
	T-2	13,783	2	6,892	1,380,188	0.000
	HT-2	3,171	2	1,586	121,198	0.000
	OTA	3,746	2	1,873	266,829	0.000
	D3G	11,103	2	5,551	45,031	0.000
	AFB1	34,447	2	17,224	2823,554	0.000
Extrusion	pH	2.038	4	0.509	2315.591	0.000
	Total titratable acidity (TTA)	14.598	4	3.650	165.886	0.000
	L(+) lactic acid	0.698	4	0.174	2024.647	0.000
	D(−) lactic acid	10.999	4	2.750	10,821.343	0.000
	LAB count	4.464	4	1.116	82.631	0.000
	Mould/Yeast	0.283	4	0.071	5.674	0.002
	Total Bacteria Count	3.104	4	0.776	45.137	0.000
	Total Enterobacteria Count	1.023	4	0.256	27.376	0.000
	Fructose	0.087	4	0.022	92.914	0.000
	Glucose	3.168	4	0.792	2121.482	0.000
	Sucrose	0.418	4	0.105	178.210	0.000
	Maltose	0.925	4	0.231	667.154	0.000
	Aspartic acid	0.001	4	0.000	0.117	0.976
	Glutamine	1.971	4	0.493	37.934	0.000
	Serine	0.049	4	0.012	19.532	0.000
	Histidine	0.010	4	0.003	25.700	0.000
	Glycine	0.031	4	0.008	12.111	0.000
	Threonine	0.013	4	0.003	5.594	0.002
	Arginine	0.051	4	0.013	18.357	0.000
	Alanine	0.048	4	0.012	24.140	0.000
	Tyrosine	0.006	4	0.001	7.944	0.000
	Cysteine	0.011	4	0.003	2.623	0.054
	Valine	0.039	4	0.010	9.423	0.000
	Methionine	0.028	4	0.007	70.700	0.000
	Tryptophan	0.030	4	0.008	8.545	0.000
	Phenylalanine	0.005	4	0.001	3.162	0.028
	Isoleucine	0.191	4	0.048	7.945	0.000
	Leucine	0.014	4	0.003	34.700	0.000
	Lysine	0.033	4	0.008	13.867	0.000
	Proline	4.617	4	1.154	352.644	0.000
	Mycotoxins					
	AOH	11,947	4	2,987	279,147	0.000
	AME	20,956	4	5,239	1,488,361	0.000
	17-DMAG	7,403	4	1,851	1,119,454	0.000
	15-DON	4,861,919	4	1,215,480	1,470,338	0.000
	MEL	0.444	4	0.111	489,265	0.000
	Neo	0.025	4	0.006	84,955	0.000
	15ACS	2,198,171	4	549,543	5,322,162	0.000
	ENN A	2,739,306	4	684,826	30,204,047	0.000
	ENN A1	167,629	4	41,907	13,150,786	0.000
	FB1	2,714	4	0.678	1,094,161	0.000
	FB2	0.004	4	0.001	147,000	0.000
	DON	4,375,972	4	1,093,993	12,732,693	0.000
	STC	23,145	4	5,786	914,584	0.000

	OTB	0.018	4	0.005	337,500	0.000
	FUSX	17,077	4	4,269	197,097	0.000
	T-2	5,421	4	1,355	271,432	0.000
	HT-2	7,708	4	1,927	147,291	0.000
	OTA	33,841	4	8,460	1,205,181	0.000
	D3G	18,670	4	4,668	37,860	0.000
	AFB1	4,083	4	1,021	167,349	0.000
LAB fermentation * Extrusion	pH	0.084	4	0.021	96.000	0.000
	Total titratable acidity (TTA)	0.582	4	0.145	6.614	0.001
	L(+) lactic acid	0.190	4	0.047	550.448	0.000
	D(−) lactic acid	0.097	4	0.024	95.287	0.000
	LAB count	5.501	8	0.688	50.909	0.000
	Mould/Yeast	1.858	8	0.232	18.625	0.000
	Total Bacteria Count	0.523	8	0.065	3.802	0.003
	Total Enterobacteria Count	2.047	8	0.256	27.376	0.000
	Fructose	0.098	8	0.012	52.350	0.000
	Glucose	0.051	8	0.006	16.929	0.000
	Sucrose	0.762	8	0.095	162.460	0.000
	Maltose	0.136	8	0.017	49.096	0.000
	Aspartic acid	0.015	8	0.002	1.734	0.131
	Glutamine	0.127	8	0.016	1.219	0.322
	Serine	0.005	8	0.001	1.058	0.417
	Histidine	0.003	8	0.000	3.950	0.003
	Glycine	0.008	8	0.001	1.547	0.183
	Threonine	0.007	8	0.001	1.491	0.202
	Arginine	0.009	8	0.001	1.686	0.143
	Alanine	0.026	8	0.003	6.410	0.000
	Tyrosine	0.003	8	0.000	1.778	0.121
	Cysteine	0.021	8	0.003	2.457	0.035
	Valine	0.023	8	0.003	2.745	0.021
	Methionine	0.004	8	0.001	5.300	0.000
	Tryptophan	0.019	8	0.002	2.733	0.022
	Phenylalanine	0.019	8	0.002	5.550	0.000
	Isoleucine	0.038	8	0.005	0.793	0.613
	Leucine	0.003	8	0.000	3.650	0.004
	Lysine	0.014	8	0.002	2.967	0.014
	Proline	1.870	8	0.234	71.398	0.000
	Mycotoxins					
	AOH	15,154	8	1,894	177,032	0.000
	AME	12,109	8	1,514	430,013	0.000
	17-DMAG	0.268	8	0.033	20,238	0.000
	15-DON	12,068,820	8	1,508,603	1,824,922	0.000
	MEL	0.057	8	0.007	31,456	0.000
	Neo	0.007	8	0.001	11,114	0.000
	15ACS	40,774	8	5,097	49,361	0.000
	ENN A	1,750,753	8	218,844	9,652,050	0.000
	ENN A1	105,407	8	13,176	4,134,678	0.000
	FB1	0.318	8	0.040	64,169	0.000
	FB2	0.008	8	0.001	147,000	0.000
	DON	1,690,544	8	211,318	2,459,474	0.000

STC	1,108	8	0.139	21,896	0.000
OTB	0.009	8	0.001	85,500	0.000
FUSX	38,377	8	4,797	221,474	0.000
T-2	16,223	8	2,028	406,119	0.000
HT-2	35,741	8	4,468	341,499	0.000
OTA	92,070	8	11,509	1,639,429	0.000
D3G	13,918	8	1,740	14,112	0.000
AFB1	8,167	8	1,021	167,349	0.000

AOH—alternariol; AME—alternariol monomethyl ether; 17-DMAG—17-dimethylaminoethylamino-17-demethoxygeldanamycin; 15-DON—15-acetyldeoxynivalenol; MEL—meleagrin; Neo—neosolaniol; 15ACS—15-acetoxyscirpenol; ENN A—enniatin A; ENN A1—enniatin A1; FB1—fumonisin B1; FB2—fumonisin B2; DON—deoxynivalenol; STC—sterigmatocystin; OTB—ochratoxin B; FUSX—fusarenon X; T-2—T-2 toxin; HT-2—HT-2 toxin; OTA—ochratoxin A; D3G—deoxynivalenol-3-glucoside; AFB1—aflatoxin B1.

Additional Information for Section Materials and Methods.

High-performance Liquid Chromatography Coupled to Tandem-Mass Spectrometry (HPLC-MS/MS) for Mycotoxin Analysis

Materials and Chemicals, Reagents, and Standards

Mycotoxin standards were all of at least 95% purity. Standards were supplied by Santa Cruz Biotechnology (Dallas, TX, USA), Romer Labs (Tulln, Austria), Fermentek (Jerusalem, Israel), Cayman Chemical Company (Ann Arbor, MI, USA). HPLC grade acetonitrile and methanol (>99% assay), ACS grade formic acid (≥96.0% assay) was used. Ultrapure water (18.2 MΩ × cm) was generated by a Milli-Q system (Millipore, Billerica, MA, USA). QuEChERS buffer-salt extraction kits consisting of magnesium sulphate (4 g), sodium chloride (1 g), trisodium citrate dihydrate (1 g), and disodium hydrogen citrate sesquihydrate (0.5 g) per portion were purchased from Phenomenex (Torrance, CA, USA). There was used two blank samples—unprocessed wheat and barley.

Chromatographic Method

The analysis was performed on an UltiMate™ 3000 (Thermo Fisher Scientific, USA) HPLC coupled with a Thermo Scientific TSQ Quantiva MS/MS detector. The separation was performed on a Phenomenex Luna C18 reversed-phase analytical column (150 × 2.0 mm, 3 μm). The autosampler was maintained at 4°C and the column temperature was 40 °C. The sample injection volume was 40 μL. Ion monitoring was conducted in both positive and negative ion modes and the mass analysis was performed in selected reaction monitoring (SRM) mode. The following instrumental settings were used: spray voltage 3.5 kV (positive ion mode), 2.5 kV (negative ion mode), vaporiser temperature 350 °C, ion transfer temperature 300 °C, sheath gas 55 arbitrary units (arb), auxiliary gas 25 arb, and sweep gas 5 arb. Data processing was performed with Xcalibur™ software (Thermo Fisher Scientific).

Six-point calibration curves were constructed using blanks spiked with mycotoxin standard mixtures (1 μg kg^{-1} (or LOQ) to 250 μg kg^{-1}). The least squares regression method was used for slope construction and calculation of the determination coefficients (R2) of the calibration curves, which were evaluated to a fit of at least 0.99.

Phase A—0.1% formic acid, 0.5 mM ammonium acetate in water; Phase B—0.1% formic acid, 0.5 mM ammonium acetate in acetonitrile.

Table. Flow gradient.

Time	Flow (mL/min)	%B	Curve
−8.00		Equilibration	
−8.00	0.350	5.0	5
−0.50	0.350	5.0	5
−0.10	0.200	5.0	5
0.00		Run	
0.00	0.200	5.0	5
30.00	0.200	99.0	5
34.95	0.200	99.0	5
36.00	0.200	99.0	5
36.00		Stop Run	

Method Duration (min): 36
Ion Source:
- Ion Source Type: H-ESI
- Spray Voltage: Static
- Positive Ion (V): 3500
- Negative Ion (V): 2500
- Sheath Gas (Arb): 55
- Aux Gas (Arb): 25
- Sweep Gas (Arb): 5
- Ion Transfer Tube Temp (°C): 300
- Vaporizer Temp (°C): 350

SRM Scan:
- Use Cycle Time: True
- Cycle Time (sec): 0.4
- Use Calibrated RF Lens: True
- Q1 Resolution (FWHM): 0.7
- Q3 Resolution (FWHM): 1.2
- CID Gas (mTorr): 1.5

Compound	Retention Time (min)	Polarity	Precursor (m/z)	Product (m/z)	Collision Energy (V)
D3G (1)	9.3	Negative	503	426.99	15
D3G (2)	9.3	Negative	503	456.97	15
DON (1)	9	Positive	297	231	12
DON (2)	9	Positive	297	249	11
FUS_X (1)	11.6	Positive	355.1	247.3	10
FUS_X (2)	11.6	Positive	355.1	277	10
NEO (2)	12.5	Positive	400.2	185	25
NEO (1)	12.5	Positive	400.2	215.1	20
MEL	14	Positive	434.5	334.1	20
MEL	14	Positive	434.5	403.2	8
15_DON (1)	13.9	Positive	339.2	137.1	15
15_DON (2)	13.9	Positive	339.2	321.2	5
3ADON (1)	14.1	Positive	339.14	203.23	15
3ADON (2)	14.1	Positive	339.14	231.1	13
17-DMAG	14.6	Positive	617.5	159.1	29
17-DMAG	14.6	Positive	617.5	345.2	29
15-acs (1)	14.7	Positive	342.3	107.1	18
15-acs (2)	14.7	Positive	342.3	265.2	10
FB1 (1)	15.4	Positive	722.5	334.25	40
FB1 (2)	15.4	Positive	722.5	352.25	35
ALT (1)	16.5	Positive	293.2	257.2	15
ALT (2)	16.5	Positive	293.2	275.2	8
FB3 (1)	16.6	Positive	706.3	318.5	37
FB3 (2)	16.6	Positive	706.3	336.3	41
T2_triol (2)	16.7	Positive	400.3	215.1	15
T2_triol (1)	16.7	Positive	400.3	281	10
FB2 (1)	17.2	Positive	706.5	318.25	38
FB2 (2)	17.2	Positive	706.5	336.2	36
Afla B1 (1)	18	Positive	313	213	43
Afla B1 (2)	18	Positive	313	241	37
Afla B1 (3)	18	Positive	313	285	23
ALX (1)	18.4	Negative	351.3	297.1	30
ALX (2)	18.4	Negative	351.3	315.1	15
AOH (1)	18.8	Negative	257	147	25
AOH (2)	18.8	Negative	257	212.9	16
HT-2 (3)	19	Positive	442.2	215.1	10
HT-2 (4)	19	Positive	442.2	263.1	10
Tentoxin (1)	19.2	Negative	413.5	141.1	24
Tentoxin (2)	19.2	Negative	413.5	271.2	15
MYR	20	Positive	402.4	320.3	25
MYR	20	Positive	402.4	384.3	10
OTB (1)	20.9	Positive	370.1	103.2	30
OTB (2)	20.9	Positive	370.1	205	20
CVD	21.8	Positive	403.2	285.2	10
CVD	21.8	Positive	403.2	297.2	10
T-2 (1)	22.2	Positive	489	327	23
T-2 (2)	22.2	Positive	489	387	21

AMEther (1)	23.1	Negative	271.3	227.1	39
AMEther (2)	23.1	Negative	271.3	256	16
OTA (1)	23.3	Positive	404	102	50
OTA (2)	23.3	Positive	404	239	25
Zea (1)	23.4	Negative	317.15	157	23.5
Zea (2)	23.4	Negative	317.15	273	18
Sterigmatocystin	24	Positive	325.1	281	36
Sterigmatocystin	24	Positive	325.1	310	24
17AAG	25	Negative	584	443	20
17AAG	25	Negative	584	541	20
Apicidin	26	Negative	622.6	462.3	18
Apicidin	26	Negative	622.6	592.4	4
ENNB (1)	31	Positive	657.4	196	32
ENNB (2)	31	Positive	657.4	214	34
ENNB1 (1)	32	Positive	671.3	196	34
ENNB1 (2)	32	Positive	671.3	214	35
ENNA1 (1)	33	Positive	699.4	210	34
ENNA1 (2)	33	Positive	699.4	228	35
ENNA (1)	34	Positive	685.2	210	31
